# Integrated serum pharmacochemistry, pharmacokinetics, and network analysis to explore active components of BuShao Tiaozhi Capsule on hyperlipidemia

**DOI:** 10.3389/fphar.2024.1444967

**Published:** 2025-01-03

**Authors:** Ruiyin Tang, Guanlin Xiao, Yanchang Liu, Dezheng Jia, Zhihao Zeng, Canchao Jia, Dongmei Li, Yangxue Li, Jieyi Jiang, Sumei Li, Xiaoli Bi

**Affiliations:** ^1^ School of the Fifth Clinical Medicine, Guangzhou University of Chinese Medicine, Guangzhou, Guangdong, China; ^2^ Guangdong Provincial Engineering Technology Research Institute of Traditional Chinese Medicine, Guangdong Provincial Key Laboratory of Research and Development in Traditional Chinese Medicine, Guangzhou, Guangdong, China

**Keywords:** BuShao Tiaozhi Capsule, serum pharmacochemistry, pharmacokinetics, network analysis, hyperlipidemia

## Abstract

BuShao Tiaozhi Capsule (BSTZC), a novel drug in China, has been used to treat hyperlipidemia (HLP) in clinical practice for many years. Despite our previous studies suggesting that BSTZC can treat HLP, there is a lack of a rapid and systematic method to explore its active components. Therefore, in this study, we aimed to investigate the active components and mechanisms of BSTZC in treating HLP by integrating serum pharmacology, pharmacokinetics, network analysis, and experimental validation. We first established UPLC fingerprints, calibrated 23 common peaks, and identified 13 common peaks, and the similarity was greater than 0.99 for 10 batches. A total of nine metabolites from BSTZC were identified in serum and considered as PK markers. The pharmacokinetic parameters of the PK markers were compared between the control group and the model group through the pharmacokinetics study to determine the dynamic changes of representative components in rats. Compared with the control group, the C_max_ and AUC_0→t_ of OXY, IVT, IVL, and KPF-3-G were significantly higher (P< 0.05); the AUC_0→∞_ of OXY, PN, and IVT was significantly higher (*P*< 0.05); and the t_1/2_ of IVT, SA, and KPF-3-G was significantly different (*P*< 0.05). *In vivo* experiments showed that BSTZC and its active components could effectively alleviate lipid metabolism disorders and liver injury, with obvious lipid-lowering effects. Further studies showed that BSTZC alleviated HLP by inhibiting the PI3K/Akt signaling pathway, which was consistent with the results of the network analysis study. Our results revealed the active components and mechanisms of BSTZC in the treatment of HLP, which could provide useful information to guide the clinical application of BSTZC.

## 1 Introduction

Hyperlipidemia (HLP) is a disorder of lipid metabolism disease manifested by an elevation in total cholesterol (TC), triglycerides (TG), and low-density lipoprotein (LDL) in blood, or decreased high-density lipoprotein (HDL), which is considered to be a major risk factor for cardiovascular diseases (Vekic et al., 2019). Hypercholesterolemia is associated with an increased risk of cardiovascular disease, and elevated plasma LDL cholesterol levels have become the eighth leading risk factor for death in 2019 (Pirillo et al., 2021). Moreover, the incidence of HLP in some developing countries has increased. At present, the common therapeutic drugs are statins and fibrates, but these drugs are often limited by undesirable side effects, such as abdominal distension, diarrhea, and myasthenia (Bahiru et al., 2021).

BuShao Tiaozhi Capsule (BSTZC) is a traditional Chinese botanical drug formula consisting of four Chinese botanical drugs, namely, Microctis Folium, Paeoniae Radix Rubra, Curcumae Rhizoma, and Andrographis Herba, in the ratio of 4.5:1.5:1:1, which has been used for the treatment of HLP in clinical practice for many years. A clinical trial for new drug approval (2016L02809) has been successfully admitted by the Nation Medical Products Administration (NMPA). It can clear heat, remove food stagnation, and invigorate blood circulation. In the previous research, we analyzed the chemical composition of BSTZC by using a UPLC-TOF-MS/MS method (Xiao et al., 2020), and the quality specification study was established at the same time. In terms of pharmacodynamic effects, BSTZC significantly reduced the serum TC, TG, and LDL-C; improved HDL-C and ApoA1/ApoB; and boosted hepatic LCAT and LXR-α gene expression in HLP rats and mice (Chen et al., 2017; Gan et al., 2018). Furthermore, the lipid-lowering mechanism of BSTZC may be related to the regulation of the FXR signaling pathway, promotion of hepatic CYP7A1 expression, inhibition of ileal bile acid negative feedback regulation, promotion of bile acid excretion, and acceleration of lipid metabolism (Xiao et al., 2022). However, the pharmacological substance basis and metabolic regulation mechanism of BSTZC’s real hypolipidemic efficacy are still unclear, which hinders the further clinical application of BSTZC.

According to the theory of serum pharmacochemistry, substances that are absorbed into the blood and reach a certain concentration are likely to be therapeutically effective (Ma, F.X. et al., 2017). In addition, the pharmacokinetics study is focused on dynamic changes and laws of the absorption, distribution, metabolism, and excretion of the effective components of drugs in the body, which was critical in novel medicine research and clinical practice (Li et al., 2015; Tang and Lu, 2009). Network pharmacology is one of the primary techniques to forecast the active components and mechanism of action of drugs, by constructing an interactive network of “disease–drug–targets pathways” (Xiao et al., 2022). Therefore, we established an integrated strategy of serum pharmacochemistry, pharmacokinetics, network analysis, and experimental validation to investigate the active components and mechanisms of BSTZC in treating HLP, which further promoted the study of the pharmacological substance basis and mechanism of BSTZC.

## 2 Materials and methods

### 2.1 Chemicals and reagents

The BSTZ (Batch number: 20,230,401) was obtained from Guangdong Provincial Second Hospital of Traditional Chinese Medicine (Guangzhou, China). The information on the reference substances is available in Supplementary Table S1. LC-MS-grade methanol, formic acid, and acetonitrile were purchased from Fisher Scientific (Fair Lawn, NJ, United States). Ultrapure water was purchased from Wahaha Co., Ltd. (Hangzhou, China). Heparin sodium (Batch No. 00321100) was purchased from Chengdu Hepatunn Pharmaceutical Co., Ltd. BSTZC was provided by the manufacturing laboratory of Guangdong Provincial Second Hospital of Traditional Chinese Medicine (Guangzhou, China). Total cholesterol (TC) kit (Batch No. 20230828) and triglyceride (TG) kit (Batch No. 20230830) were purchased from Nanjing Jiancheng Bioengineering Institute (Nanjing, China). A high-fat diet (52.2% standard diet, 0.2% bile sodium, 10% casein, 1.2% cholesterol, 0.6% calcium hydrogen phosphate, 0.4% mineral feed, 0.4% premix feed, 15% lard, and 20% saccharose) was purchased from Guangdong Medical Laboratory Animal Center.

### 2.2 Animal

The Guangdong Medical Laboratory Animal Center (Guangdong Provincial Engineering Technology Research Institute of Traditional Chinese Medicine) SPF Animal Lab provided SD male rats (220 ± 20 g) with license number SCXK (Yue) 2022–0002 and C57BL/6 male mice (18∼22 g) with license number SCXK (Yue) 2022–0059. The Animal Ethics Committee of the Guangdong Medical Laboratory Animal Center (Guangdong Provincial Engineering Technology Research Institute of Traditional Chinese Medicine) permitted them to carry out experiments (Approval No. 049097). SD rats were maintained for 1 week in an SPF-grade laboratory with a 12-h light/dark cycle.

### 2.3 UPLC fingerprint analysis of BSTZ

#### 2.3.1 Sample preparation

The BSTZ was pulverized and 2.0 g of the powder was dissolved in 25 mL of 75% methanol–water solution and extracted by sonication for 30 min. The extract was centrifuged and the supernatant was filtered through a 0.22-μm membrane for use. All 10 batches of BSTZ were prepared using the same procedure to facilitate subsequent analysis.

In addition, appropriate quantities of standard samples were weighed accurately. These standard samples were dissolved in 75% methanol and diluted to 10 mL, and the supernatant was filtered through a 0.22-μm filter and used as the reference solution for UPLC analysis.

#### 2.3.2 Chromatographic conditions for UPLC fingerprint

BSTZ analysis was performed on a UPLC (2,695, Waters, United States) connected with a CORTECS UPLC C18 column (2.1 × 150 mm, 1.6 µm, Waters, United States). Methanol (A) and 0.1 formic acid aqueous solvent (B) were used as the mobile phase with a flow rate of 0.20 mL/min, and the solvent gradient used was as follows: 0∼35 min, 7∼26% A; 35∼50 min, 26%∼26% A; 50∼60 min, 26%∼35% A; 60∼80 min, 35%∼40% A; and 80∼90 min, 40%∼60% A. The volume temperature was set at 35°C, and the injection volume was 1.0 µL. The result was detected at 300 nm.

### 2.4 Serum pharmacochemistry

#### 2.4.1 Preparation of sample solution

The contents of BSTZC (about 0.5 g) were precisely weighed and added with 25 mL 75% methanol–water mixture, and then ultrasonically extracted at room temperature for 30 min. Following the extraction process, the mixture was centrifuged at 12,000 rpm for 10 min. The supernatant was filtered through a 0.22-µm Millipore filter before the analysis was performed.

#### 2.4.2 Preparation of reference component solutions

A certain amount of 25 reference components was prepared individually by dissolving each component in methanol at a certain concentration. The stock solutions were diluted and mixed with methanol to obtain standard solutions at a concentration of roughly 50 μg/mL for each component. The standard solutions were filtered through Millipore filters with a pore size of 0.22 µm before the analysis was performed.

#### 2.4.3 Animal experiment and preparation of plasma sample

Twelve male SD rats (220 ± 20 g) were purchased from Guangdong Medical Laboratory Animal Center (Certificate number 44007200115140). They were given access to normal laboratory food and water for 1 week at a time, and then they were randomly divided into a control group and an administration group. Subjects abstained from food for a duration of 12 h before the experiment and water was provided without restriction. The administration group rats received the human equivalent dose of BSTZC at a dose of 5.76 g/kg, and the control group rats were given an equal dose of water intragastrically. At 10 min, 20 min, 30 min, 60 min, 90 min, and 120 min after administration, blood samples were collected from the fundus venous plexus and centrifuged at 3,000 rpm at 4°C for 15 min. Then, the supernatants of the rats were combined equally at each time point. All blood samples obtained were frozen at −80°C before analysis.

Protein was precipitated by adding 4 mL of methanol to a 1 mL mixture and vortexing for 30 s. After placing at −20°C for 1 h, the mixture was centrifuged at 12,000 rpm at 4°C for 15 min. The supernatant was removed and blown dry at room temperature in nitrogen. The residue was redissolved in 150 μL methanol and vortex mixed for 30 s, and then centrifuged at 12,000 rpm and 4°C for 15 min. Afterward, 90 μL of supernatant was produced for UPLC-Q-TOF-MS analysis.

#### 2.4.4 Chromatography conditions and mass spectrometry conditions

Chromatography analyses were performed on Agilent 1,290Ⅱ (Agilent, United States). A Waters ACQUITY UPLC BEH C18 column (100 × 2.1 mm, 1.7 μm) was used to separate and analyze samples. Eluent A was 0.1% formic acid water solution, whereas eluent B was acetonitrile with a flow rate of 0.3 mL/min and a temperature of 30°C. The gradient elution was as follows: 0–3 min, 10% B; 3–10 min, 10%–20% B; 10–15 min, 20%–30% B; 15–23 min, 30%–50% B; 23–30 min, 50%–68% B; 30–34 min, 68%–80% B; 34–40 min, 80%–95% B; 40–45 min, 95%–5% B; and 45–50 min, 5% B. The injection volume was 1 μL.

The MS analyses were performed on an X500 Q-TOF/MS system (AB Sciex, United States). The optimized operating parameters were as follows: ion source, 379 KPa; curtain gas, 241.3 KPa; the full scan mass range, *m/z*100-1,000; and ion source temperature, 500°C. The mass spectrometer analysis was conducted in both positive and negative ion modes with an electrospray interface (ESI) source. The capillary voltage was 5.5 KV, the declustering potential was 100 V, and collision energy is 35 eV in the ESI^+^ mode. The capillary voltage was −4.5 KV, the declustering potential was −80 V, and collision energy is 35 eV in the ESI^−^ mode. Natural Products HR-MS/MS Spectral Library (Version 1.0, AB Sciex, United States) in Sciex OS v2.1 software was used to analyze the component data. The serum migrating metabolites of BSTZC then were regarded as PK markers.

### 2.5 Pharmacokinetics study

#### 2.5.1 Preparation of standards and control samples

The standards of oxypaeoniflorin (OXY), paeoniflorin (PN), isovitexin (IVT), isoviolanthin (IVL), salicylic acid (SA), kaempferol-3-glucuronide (KPF-3-G), narcissoside (NCS), apigenin-7-glucuronide (APG-7-G), and neoandrographolide (NAG) were accurately weighed and dissolved in methanol to obtain the stock solution. Then, they were diluted with methanol to make mixed standard solution with concentrations of 6.360 μg/mL, 4.873 μg/mL, 6.460 μg/mL, 19.646 μg/mL, 60.329 μg/mL, 11.152 μg/mL, 9.813 μg/mL, 24.084 μg/mL, and 24.132 μg/mL. The stock solution of sulfamethoxazole was accurately weighed, dissolved, and diluted with methanol at a concentration of 2.011 μg/mL.

#### 2.5.2 Sample preparation

A centrifuge tube containing 100 μL plasma was filled with 400 μL methanol–acetonitrile mixed solution (1:1) and IS solution (2.011 μg/mL), and then vortex mixed for 30 s and stored at −20°C for 1 h. The supernatant was separated and dried under a stream of nitrogen at room temperature after the frozen mixture was centrifuged at 4°C and 12,000 rpm for 15 min. The residue was reconstituted in 100 μL of methanol–water (80:20, v/v), vortex mixed, and centrifuged at 4°C, 12,000 rpm for 15 min, and 80 μL supernatant was taken for analysis.

#### 2.5.3 UPLC-MS/MS conditions for pharmacokinetics

Agilent 1290Ⅱ (Agilent, United States) was used to analyze plasma on an Agilent Extend-C18 RRHD column (50 × 2.1 mm, 1.8 μm) at 35°C. The mobile phase A and phase B, respectively, were 0.1% formic acid water solution and acetonitrile. The gradient was optimized as follows: 0∼0.5 min, 10% B; 0.3∼3 min, 10%∼16% B; 3∼6 min, 16% B; 6∼8 min, 16%∼20% B; 8∼9.5 min, 20%∼38% B; 9.5∼10.5 min, 38%∼95% B; and 10.5∼13 min, 95% B. The flow rate was 0.3 mL/min and the injection volume was 1 μL. Samples were analyzed in negative ionization with the MRM mode in Agilent 6495C (Agilent, United States). The main mass spectral parameters were set as follows: the capillary voltage was 3.0 KV, the ion source temperature was 500°C, the drying gas temperature was 200°C with a gas flow rate of 15 mL/min, the ion source was 30 psi, and sheath gas was 350°C with a gas flow rate of 11 mL/min. The specific mass spectrometry parameters of analytes and IS are shown in Supplementary Table S2.

#### 2.5.4 Method validation

Specificity test: selectivity was assessed by comparing the MRM chromatograms of blank plasma samples, blank plasma spiked with standards, blank solution, and representative plasma samples after oral administration, mixed standard solution, and blank solution were used for the analysis. Linearity and LLOQ: the calibration curves for the quantitative evaluation were determined by graphing the peak area ratio (y) of each component to IS versus the nominal concentration (x) by using (1/x^2^) least-squares linear regression. In addition, the analytical signaling of the LLOQ sample should be at least 10 times the signaling of the blank sample. Accuracy and precision test: the QC samples at low, middle, and high concentration levels (n = 6) were analyzed for variation and precision (intra-day and inter-day). Stability test: stability was evaluated by analyzing three concentrations of QC samples (n = 6) under four storage conditions, including room temperature for 24 h, 4°C for 12 h, three cycles of freeze-thaw at −20°C and −60°C for 30 days. Extraction recovery and matrix effect: the peak area of pre-extracted QC samples was recorded as A and the peak area of post-extracted QC samples was recorded as B. Extraction recovery = (B/A) × 100%. The peak area of mixed standard solution with IS was recorded as C. Matrix effect = (C/A) × 100%.

#### 2.5.5 Animal experiment and data analysis

Sixteen male SD rats (220 ± 20 g) were purchased from Guangdong Medical Laboratory Animal Center (Certificate number 44007200122329). They were randomly divided into two groups (n = 8): the control group was administered with a basal diet and the HLP model group was fed with a high-fat diet for 4 weeks. Four weeks after modeling, the levels of TC and TG in plasma were measured to evaluate the model efficiency. Two assays were performed by kit instructions. Then, the livers of three rats in each group were stained with hematoxylin and eosin, combined with TC and TG levels, to evaluate whether the HLP rat model was successfully established.

After the model’s successful establishment, all rats were starved overnight before the formal trial. Two group rats were administered BSTZC at a dose of 5.76 g/kg. Blood samples (0.5 mL) were collected from the fundus venous plexus and placed into heparinized tubes before the experiment and at 0, 0.083, 0.167, 0.333, 0.146, 0.5, 1, 1.5, 2, 3, 4, 6, 8, and 12 h after administration. The supernatant was obtained after centrifugation at 10,000 rpm and 4°C for 15 min. All blood samples were stored at −80°C until analysis.

The pharmacokinetic parameters were calculated by non-compartmental analysis using Phoenix WinNonlin 8.1 software, including the elimination half-time (t_1/2_), time to the maximum concentration (T_max_), maximum concentration (C_max_), area under the plasma concentration-time curve (AUC), and mean retention time (MRT).

### 2.6 Network analysis

The PK markers of BSTZC were input into the PubChem database (https://pubchem.ncbi.nlm.nih.gov/) to download Canonical SMILES, which could be used to discover the interrelated targets in the Swiss Target Prediction databases (http://www.swisstargetprediction.ch/) and then the target names were standardized from the UniProt database (https://www.uniprot.org/). In the meantime, HLP-related genes were collected from CTD (http://ctdbase.org/) and GeneCards (http://www.genecards.org/) databases, with “hyperlipidemias” and “hyperlipidemia” as keywords. To find out the overlapped targets between the component targets and disease targets, Venny 2.1.0 (http://bioinfogp.cnb.csic.es/tools/venny/) was used. The common targets were imported into the String database (http://string-db.org/), with the screening condition “*Homo sapiens*.” Cytoscape 3.7.1 software is used to visualize the protein–protein (PPI) network. Finally, we imported the targets into Bioinformatics (https://www.bioinformatics.com.cn/) to conduct the Kyoto Encyclopedia of Genes and Genomes (KEGG) data obtained pathway enrichment.

### 2.7 Lipid-lowering experimental validation of active components

#### 2.7.1 Experimental design

The acute HLP model in mice was induced by triton WR-1339 using our previously described method (Xiao et al., 2023). Male C57BL/6 mice were acclimated and fed for 1 week and then randomly divided into 13 groups of six mice each: control, TWR model, fenofibrate (26 mg/kg/day), BSTZC (4.16 g/kg/day), APG (26 mg/kg/day), IVT (26 mg/kg/day), IVL (26 mg/kg/day), KPF (26 mg/kg/day), NCS(26 mg/kg/day), NAG(26 mg/kg/day), OXY (26 mg/kg/day), PN(26 mg/kg/day), and SA (26 mg/kg/day). The animal experiment procedure is shown in Supplementary Figure S1.

#### 2.7.2 Biochemical analysis

The levels of TC, TG, LDL-c, ALT, and AST in serum were determined by using commercial kits according to the manufacturer’s instructions.

#### 2.7.3 Real-time PCR

TRIzol reagent was used to extract total RNA from each group of liver tissues. The M-MuLV First Strand cDNA Synthesis Kit and the 2X SG Fast qPCR Master Mix were utilized for reverse transcription and RT-qPCR, respectively. RT-qPCR was performed with the manufacturer’s experimental instructions by StepOnePlus (United States), and the gene primer sequences used are shown in Supplementary Table S3. The mRNA expression levels of genes were normalized to GAPDH.

### 2.8 Statistical analysis

All data were expressed as mean ± SD. Comparisons between multiple groups were evaluated using one-way ANOVA with Tukey’s multiple comparison test and analyzed in the study using GraphPad Prism 9.0 software, with statistical significance denoted as *p* < 0.05.

## 3 Results

### 3.1 Establishment of fingerprint profiles and similarity evaluation

**FIGURE 1:**
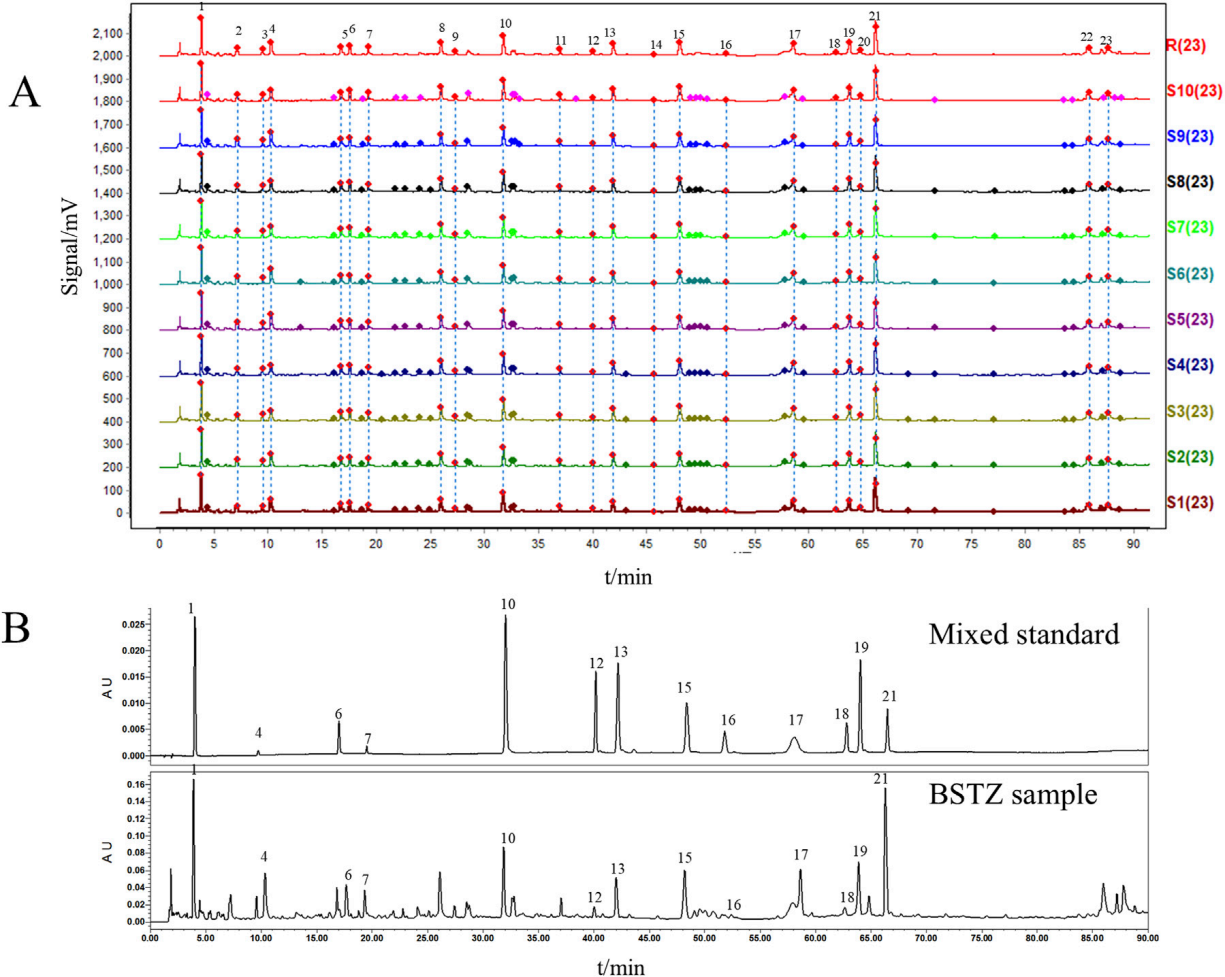
BSTZ quality control. **(A)** UPLC fingerprints of BSTZ samples from 10 batches and controls. **(B)** A total of 13 peaks were identified by standards. Peaks 1, 4, 6, 7, 10, 12, 13, 15, 16, 17, 18, 19, and 21 were gallic acid, oxypaeoniflorin, chlorogenic acid, paeoniflorin, ferulic acid, salicylic acid, vitexin, isovitexin, neoandrographolide, isoviolanthin, astragalin, narcissoside, and neoandrographolide.

UPLC prepared and analyzed 10 batches of BSTZ samples. A total of 23 common peaks were obtained based on multipoint correction (Figure 1A). In addition, the UPLC fingerprints of 10 batches of BSTZ samples were evaluated for similarity, and the similarity was more than 0.99 (Supplementary Table S4). This indicates that the quality of the BSTZ samples has been stable. Thirteen peaks, namely, gallic acid, oxypaeoniflorin, chlorogenic acid, paeoniflorin, ferulic acid, salicylic acid, vitexin, isovitexin, neoandrographolide, isoviolanthin, astragalin, narcissoside, and neoandrographolide, were then identified using standard substances (Figure 1B).

### 3.2 Identification of absorbed metabolites of BSTZC

**FIGURE 2:**
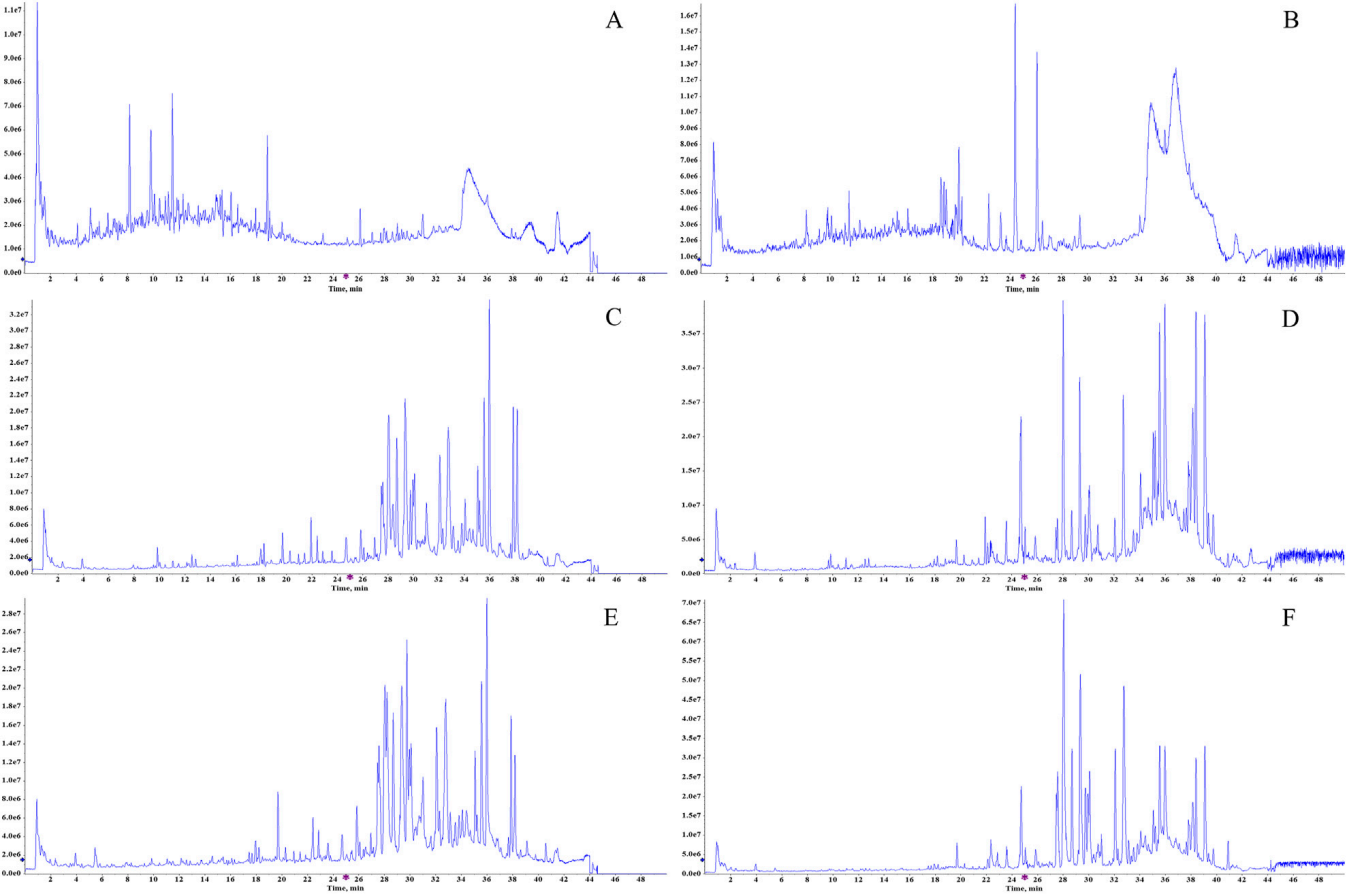
Total ion chromatograms of the BSTZC in the positive/negative ion mode **(A, B)**, blank serum in the positive/negative ion mode **(C, D)**, and rat serum after oral administration of BSTZC in the positive/negative ion mode **(E, F)**.

A total of 62 components of BSTZC were identified by comparison with standards, mass-to-charge ratio of fragment ions, and internal databases, consisting mainly of 27 flavonoids, 16 organic acids, five monoterpenoids, six diterpene lactones, and eight sesquiterpenes (Figure 2; Supplementary Table S5). Chemical profiling of serum obtained after BSTZC administration was performed to characterize the absorbed metabolites to find possible therapeutic components of BSTZC. Upon comparison with blank serum, nine metabolites were detected in the serum of healthy rats after the administration of BSTZC (OXY, PN, IVT, IVL, SA, NCS, NAG, KPF-3-G, and APG-7-G) (Figure 2; Supplementary Table S5). The two metabolites (KPF-3-G and APG-7-G) were converted from flavonoids through phase Ⅱ metabolic reaction *in vivo* to glycolaldehyde products.

### 3.3 Pharmacokinetics study

#### 3.3.1 Method validation

**FIGURE 3:**
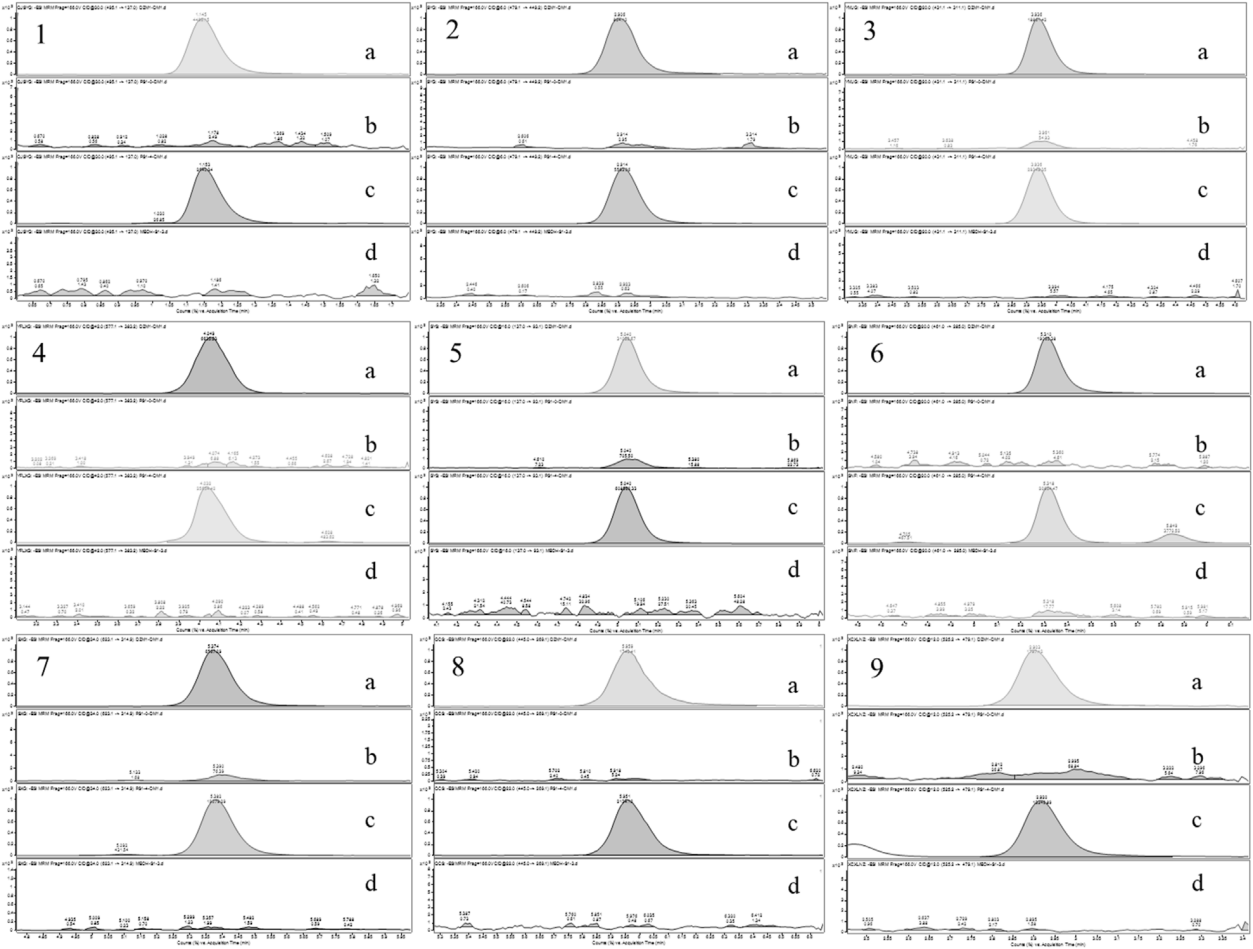
Chromatograms of nine tested components (**(A)** mixed standard solution, **(B)** blank plasma, **(C)** serum sample after oral administration of BSTZC, and **(D)** blank solution. 1: OXY; 2: PN; 3: IVT; 4: IVL; 5: SA; 6: KPF-3-G; 7: NCS; 8: APG-7-G; 9: NAG).

Based on the UPLC-MS technology, a multi-component analytical method was successfully established and methodologically investigated for the analysis of BSTZC in rat plasma. The typical MRM chromatograms of OXY, PN, IVT, IVL, SA, KPF-3-G, NCS, APG-7-G, and NAG are shown in Figure 3. The results presented show that endogenous chemicals did not interfere with the detection of the targeted components in rat plasma and internal standard, indicating the method had a good level of sensitivity and the instrument had adequate distinctiveness. The linear equation, linear ranges, coefficients (r), and LLOQs of these nine analytes are represented in Table 1. These results confirmed that the method was sufficiently sensitive for the pharmacokinetics study. The precision and accuracy values of the intra-day and inter-day of nine analytes are listed in Table 2. The results indicated that the precision and accuracy of the method were acceptable to the requirements of the biological samples. The stability result of the nine components is summarized in Table 3. The RSD of nine analytes was less than 15%, and the RE ranged from −15% to 15%, demonstrating that all the analytes were stable in the rat plasma under the four conditions, including room temperature for 24 h, 4°C for 12 h, three cycles of freeze-thaw at −20°C, and −60°C for 30 days. As shown in Table 4, the extraction recoveries and matrix effects were, respectively, 94.98%–114.70% and 85.00%–109.13%, confirming that the extraction recoveries were reliable and the plasma matrix did not interfere with the determination of the analytes.

**TABLE 1:** Regression equations, linear ranges, correlation coefficients, and LLOQs of analytes.

Component	Calibration curve	R	Range (ng/mL)	LLOQ (ng/mL)
OXY	*Y* = 0.245983*X*+0.001045	0.9958	0.0894∼496.86	0.1789
PN	*Y* = 0.0034332*X*+0.00094	0.9984	0.7309∼4,060.77	1.4619
IVT	*Y* = 1.358451*X−*0.020610	0.9979	0.0908∼504.70	0.1817
IVL	*Y* = 0.433634*X+*0.013252	0.9995	0.3613∼2007.04	0.5658
SA	*Y* = 2.208291*X*−0.028258	0.9950	0.9049∼5,027.40	1.8099
KPF-3-G	*Y* = 0.797102*X*−0.031415	0.9978	0.1613∼896.11	0.3226
NCS	*Y* = 0.642029*X−*0.014476	0.9981	0.1413∼785.02	0.3226
APG-7-G	*Y* = 0.808981*X*+0.000439	0.9963	0.3613∼2010.96	0.7239
NAG	*Y* = 0.093739*X*−0.000172	0.9958	0.3612∼2007.04	0.7225

**TABLE 2:** Regression equations, linear ranges, correlation coefficients, and LLOQs of analytes (n = 6).

Component	Concentration (ng/mL)	Precision (RSD%)		Accuracy (RE%)	
		Intra-day	Inter-day	Intra-day	Inter-day
OXY	0.60	9.92	11.57	−1.15	3.58
	5.96	8.02	10.65	−0.15	5.90
	397.49	4.95	6.36	9.56	12.92
PN	4.87	9.46	9.91	−9.78	−12.26
	48.72	6.72	10.28	−5.70	−6.98
	3,248.62	10.94	13.66	7.37	−1.79
IVT	0.61	11.32	12.45	−5.50	−5.32
	6.06	6.35	6.33	−0.54	−1.72
	403.76	4.89	6.31	10.07	12.31
IVL	1.89	8.89	10.19	−6.80	−7.51
	18.86	4.27	5.33	11.53	−13.86
	1,257.34	8.53	9.29	12.15	13.00
SA	6.03	8.92	10.94	2.02	−1.03
	60.03	7.35	7.12	5.53	6.36
	4,021.92	3.84	6.73	9.63	12.35
KPF-3-G	1.08	7.75	9.60	6.15	−5.45
	10.75	7.13	9.00	−2.84	−3.57
	716.89	3.13	8.58	−1.18	−1.71
NCS	0.94	13.41	13.10	−3.51	−6.73
	9.42	4.92	5.62	7.62	8.87
	628.02	9.73	9.56	2.27	−0.33
APG-7-G	2.41	10.59	11.80	0.62	−1.74
	24.08	9.08	9.52	−3.10	−6.68
	1,650.63	9.47	9.94	3.81	6.70
NAG	2.41	8.33	10.75	−6.65	−10.21
	24.13	7.64	8.16	5.80	9.06
	1,608.77	10.93	10.61	1.96	4.75

**TABLE 3:** Stability of results (n = 6).

Component	Concentration	24°C, 24 h	4°C, 12 h			Three cycles of freeze-thaw		−60°C, 30 d	
	(ng/mL)	RE (%)	RSD (%)	RE (%)	RSD (%)	RE (%)	RSD (%)	RE (%)	RSD (%)
OXY	0.60	3.31	11.82	−0.90	14.26	3.14	10.32	10.01	14.74
	5.96	14.65	8.02	11.29	8.18	9.54	7.54	2.93	4.06
	397.49	13.21	7.63	13.69	4.99	9.17	4.95	5.93	6.75
PN	4.87	−13.80	10.59	−12.13	12.15	−7.57	13.04	−0.16	3.82
	48.72	−5.98	12.97	−11.62	8.58	−6.49	11.63	−7.66	12.62
	3,248.62	2.24	14.23	2.87	11.79	4.17	11.10	1.08	12.85
IVT	0.61	−11.20	9.02	−13.20	11.43	−8.43	12.84	−1.23	10.00
	6.06	−2.43	6.12	0.44	9.04	−4.38	6.71	−8.78	2.74
	403.76	13.32	10.08	8.59	4.97	9.77	4.95	2.42	6.49
IVL	1.89	−10.12	13.07	−10.08	10.57	13.58	13.07	4.65	11.21
	18.86	−14.41	7.49	−11.41	5.93	−14.39	7.58	−14.47	5.88
	1,257.34	9.94	9.83	0.76	4.44	−2.90	5.93	−2.95	7.04
SA	6.03	−0.33	11.76	3.71	12.33	−4.29	13.40	−7.96	13.23
	60.03	8.00	7.17	10.43	10.24	10.99	7.34	6.43	1.94
	4,021.92	13.90	10.03	6.98	5.16	1.34	5.48	1.86	6.56
KPF-3-G	1.08	−12.42	7.04	−7.42	14.36	−10.49	11.71	−2.05	7.69
	10.75	−3.21	12.65	−0.70	8.44	−12.42	13.41	−14.58	14.14
	716.89	−4.42	10.37	−5.24	9.01	−3.53	14.97	−8.46	9.67
NCS	0.94	−13.46	11.64	1.90	13.79	3.32	12.97	−10.66	10.99
	9.42	10.00	7.83	11.92	8.40	−8.17	6.32	−9.24	13.12
	628.02	−1.21	10.43	0.08	9.56	0.04	14.05	−5.30	10.18
APG-7-G	2.41	−2.04	7.40	0.76	6.60	−6.79	7.24	−7.57	9.77
	24.08	−5.29	7.76	−2.08	6.29	−3.32	7.20	−8.56	2.06
	1,650.63	12.07	10.65	−1.67	4.50	−5.37	5.86	−8.38	6.71
NAG	2.41	−12.83	12.79	−14.69	9.72	−12.84	12.51	−10.73	9.97
	24.13	8.79	7.26	8.70	10.08	6.98	8.12	−0.58	2.32
	1,608.77	4.81	12.64	8.71	5.20	−13.19	3.82	−12.30	6.30

**TABLE 4:** Extraction recovery and matrix effects (n = 6).

Component	Concentration (ng/mL)	Extraction recovery		Matrix effect	
		Mean (%)	RSD (%)	Mean (%)	RSD (%)
OXY	0.60	103.26	11.59	90.60	12.38
	5.96	114.70	3.18	85.00	1.37
	397.49	110.10	5.27	88.19	6.30
PN	4.87	94.98	7.28	100.19	7.12
	48.72	98.26	2.26	82.43	3.42
	3,248.62	97.04	5.51	89.31	4.74
IVT	0.61	99.84	4.08	96.62	4.53
	6.06	107.28	3.05	99.83	2.67
	403.76	103.49	5.91	97.96	2.07
IVL	1.89	103.79	8.23	99.94	11.96
	18.86	110.90	2.13	109.13	3.37
	1,257.34	112.36	3.00	103.53	3.49
SA	6.03	104.83	5.80	107.85	6.13
	60.03	113.02	1.70	97.18	1.54
	4,021.92	106.58	1.99	104.98	2.68
KPF-3-G	1.08	100.17	4.62	87.75	10.39
	10.75	108.17	3.91	85.24	1.66
	716.89	105.37	1.96	87.75	3.61
NCS	0.94	101.66	6.65	87.08	8.75
	9.42	102.61	2.45	86.39	4.76
	628.02	98.78	3.51	92.33	9.01
APG-7-G	2.41	108.48	5.94	103.94	2.73
	24.08	112.26	6.12	98.47	9.13
	1,650.63	100.60	1.17	91.54	2.99
NAG	2.41	100.30	5.72	99.71	8.14
	24.13	108.33	6.10	92.76	9.03
	1,608.77	101.63	1.45	93.71	5.73

#### 3.3.2 Evaluation of the HLP rat model

**FIGURE 4:**
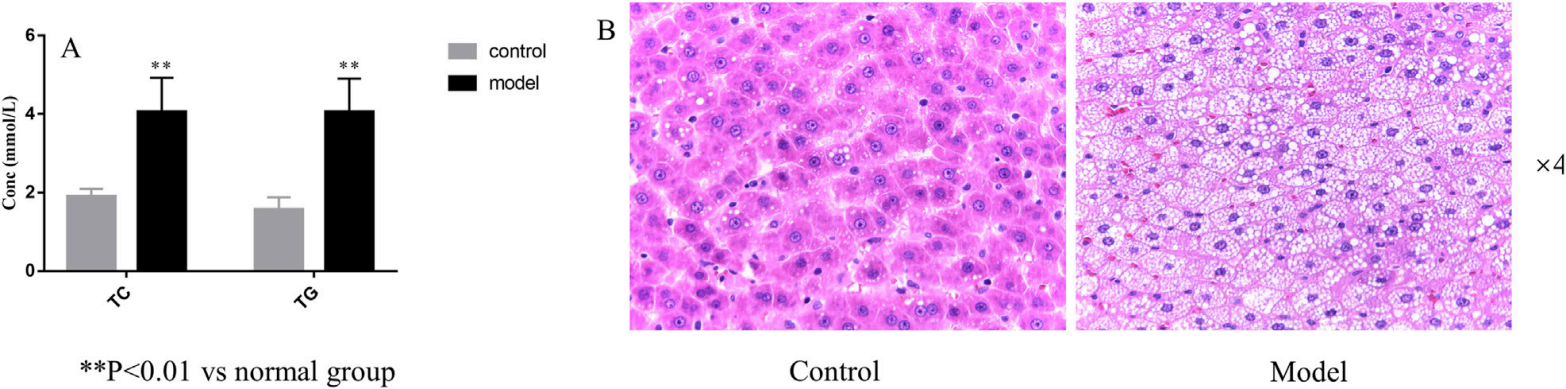
Serum biochemical parameters **(A)** and HE staining of liver sections **(B)**.

As shown in Figure 4A, the levels of TC and TG in the model group were significantly increased compared with those in the control group (*P*< 0.01). As shown in Figure 4B, the volume of liver cells in the model group was larger than that in the normal group. In the HLP group, a large number of fat vesicles appeared in the liver tissue, and the hepatocytes were balloon shaped. Meanwhile, many lipid droplets of different sizes accumulated seriously in the hepatocytes, resulting in fatty liver symptoms. The above indicated that the model was constructed successfully.

#### 3.3.3 Pharmacokinetic comparison of BSTZC in control rats and HLP model rats

**FIGURE 5:**
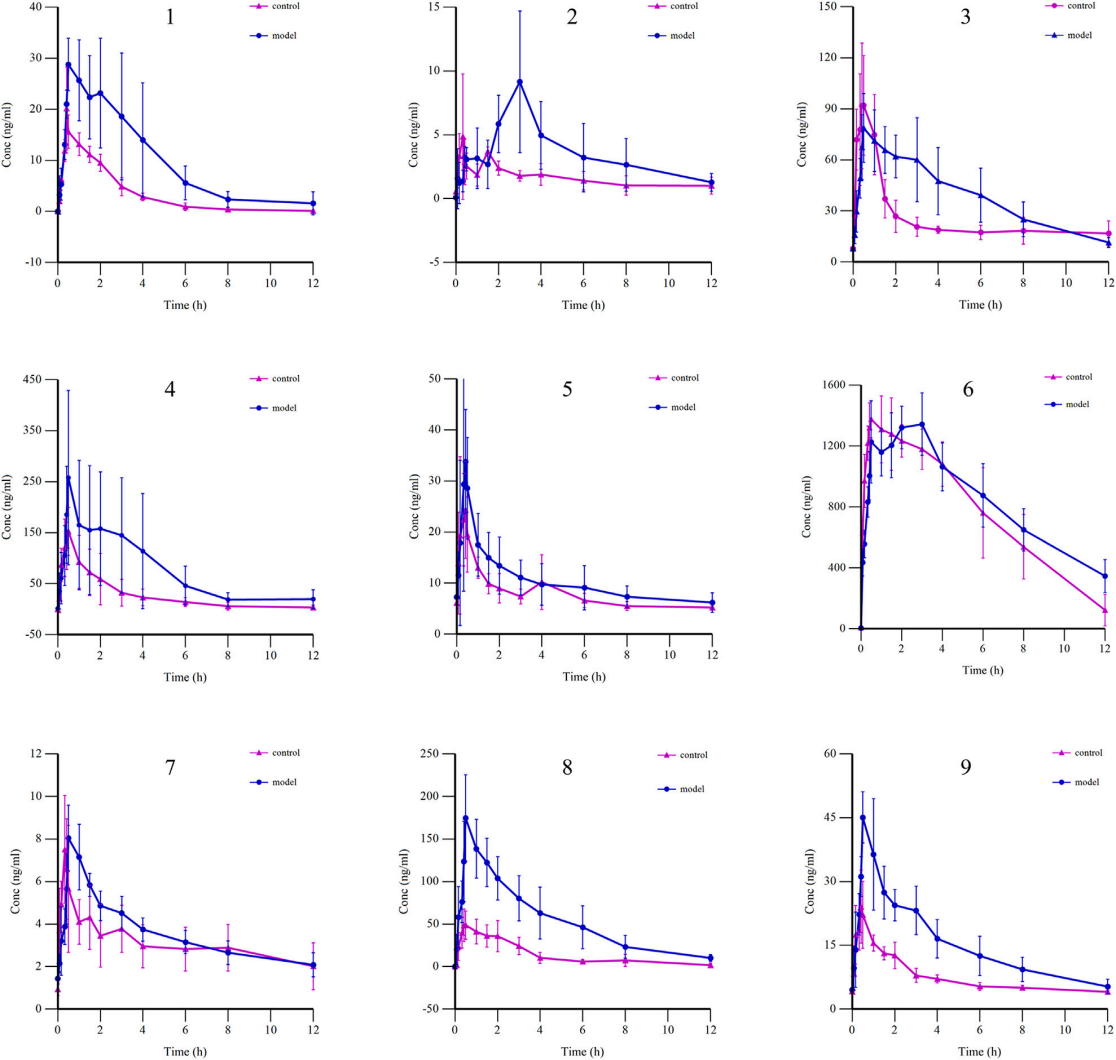
Mean plasma concentration–time curves of nine components in control and HLP model groups (1: OXY; 2: PN; 3: IVT; 4: IVL; 5: SA; 6: KPF-3-G; 7: NCS; 8: APG-7-G; 9: NAG).

The mean plasma concentration–time curves of nine components in the control and the HLP model group are shown in Figure 5, whereas the main pharmacokinetic parameters are shown in Table 5. Compared with the control group, mostly the C_max_ and AUC were higher in the model group, whereas the C_max_ and AUC_0→t_ of OXY, IVT, IVL, and KPF-3-G were significantly higher in the model group (*P*< 0.05), and the AUC_0→∞_ of OXY, PN, and IVT was significantly higher in the model group (*P*< 0.05). T_max_ reflected the rate of drug absorption. T_max_ parameters were not significantly different between the model group and the normal group except for OXY, IVT, SA, and APG-7-G. On the other hand, the parameters of t_1/2_ and MRT were crucial factors in drug metabolism and elimination. As the result shows, most of the components’ MRT were less than 6 h. Moreover, the t_1/2_ of IVT, SA, and KPF-3-G was significantly different (*P*< 0.05).

**TABLE 5:** Main pharmacokinetic parameters of nine analytes (x̄±s, n = 6).

Component	Group	t_1/2_ (h)	T_max_ (h)	C_max_ (ng/mL)	AUC_0→t_ (h^*^ng/mL)	AUC_0→∞_ (h^*^ng/mL)	MRT_0→t_ (h)
OXY	Control	1.39 ± 0.55	0.45 ± 0.05	21.66 ± 7.03	39.51 ± 7.43	40.41 ± 7.18	2.03 ± 0.44
	Model	2.16 ± 1.03	1.00 ± 0.61^*^	33.10 ± 5.36^*^	115.01 ± 51.02^*^	122.52 ± 46.68^*^	3.22 ± 0.47^*^
PN	Control	1.91 ± 1.12	0.48 ± 0.04	152.52 ± 46.99	326.56 ± 145.09	344.08 ± 152.29	2.48 ± 0.75
	Model	3.55 ± 2.46	1.08 ± 1.10	275.50 ± 148.19	893.99 ± 632.57	1,042.31 ± 584.15^*^	3.59 ± 1.12
IVT	Control	10.21 ± 6.11	0.33 ± 0.16	27.43 ± 5.90	90.23 ± 9.18	150.43 ± 42.40	4.35 ± 0.22
	Model	5.16 ± 1.54^**^	0.60 ± 0.22^*^	48.38 ± 7.83^**^	183.12 ± 29.55^**^	222.56 ± 42.67^*^	4.08 ± 0.41
IVL	Control	2.62 ± 1.84	0.48 ± 0.04	54.70 ± 18.44	164.59 ± 47.19	180.68 ± 54.88	4.10 ± 2.24
	Model	3.37 ± 1.35	0.60 ± 0.22	184.60 ± 41.25^**^	643.59 ± 182.36^**^	692.49 ± 186.06^**^	3.53 ± 0.49
SA	Control	2.13 ± 0.57	0.67 ± 0.31	1,472.74 ± 80.94	9,232.85 ± 1903.57	9,668.54 ± 2,343.94	4.13 ± 0.44
	Model	5.15 ± 2.53^*^	2.30 ± 0.67^**^	1,425.11 ± 175.82	10,152.94 ± 1,461.59	12,909.30 ± 2,666.50	4.76 ± 0.15^*^
KPF-3-G	Control	11.04 ± 3.23	0.38 ± 0.14	110.61 ± 12.48	304.41 ± 49.22	577.34 ± 219.04	4.39 ± 0.69
	Model	4.25 ± 1.57^*^	1.00 ± 1.11	86.62 ± 17.37^*^	462.80 ± 116.83^*^	533.89 ± 101.16	6.39 ± 1.41^*^
NCS	Control	10.07 ± 5.07	0.35 ± 0.12	33.73 ± 7.70	95.38 ± 13.80	172.36 ± 51.07	4.77 ± 0.32
	Model	15.46 ± 10.72	0.42 ± 0.07	45.38 ± 8.66	122.94 ± 32.61	251.42 ± 67.04	4.65 ± 0.38
APG-7-G	Control	12.35 ± 12.22	0.52 ± 0.56	6.49 ± 4.05	19.25 ± 3.88	46.12 ± 33.46	4.61 ± 1.15
	Model	4.06 ± 1.38	2.28 ± 1.13^**^	9.80 ± 4.62	42.44 ± 20.35	50.19 ± 23.73	4.56 ± 0.79
NAG	Control	9.95 ± 5.51	0.38 ± 0.07	9.08 ± 1.73	37.51 ± 9.94	72.54 ± 38.77	5.06 ± 0.42
	Model	10.80 ± 3.28	0.60 ± 0.22^*^	8.14 ± 1.41	42.61 ± 4.07	76.84 ± 23.09	4.78 ± 0.37

* P< 0.05, ^**^ *P*< 0.01 vs. normal group.

#### 3.3.4 Network analysis

**FIGURE 6:**
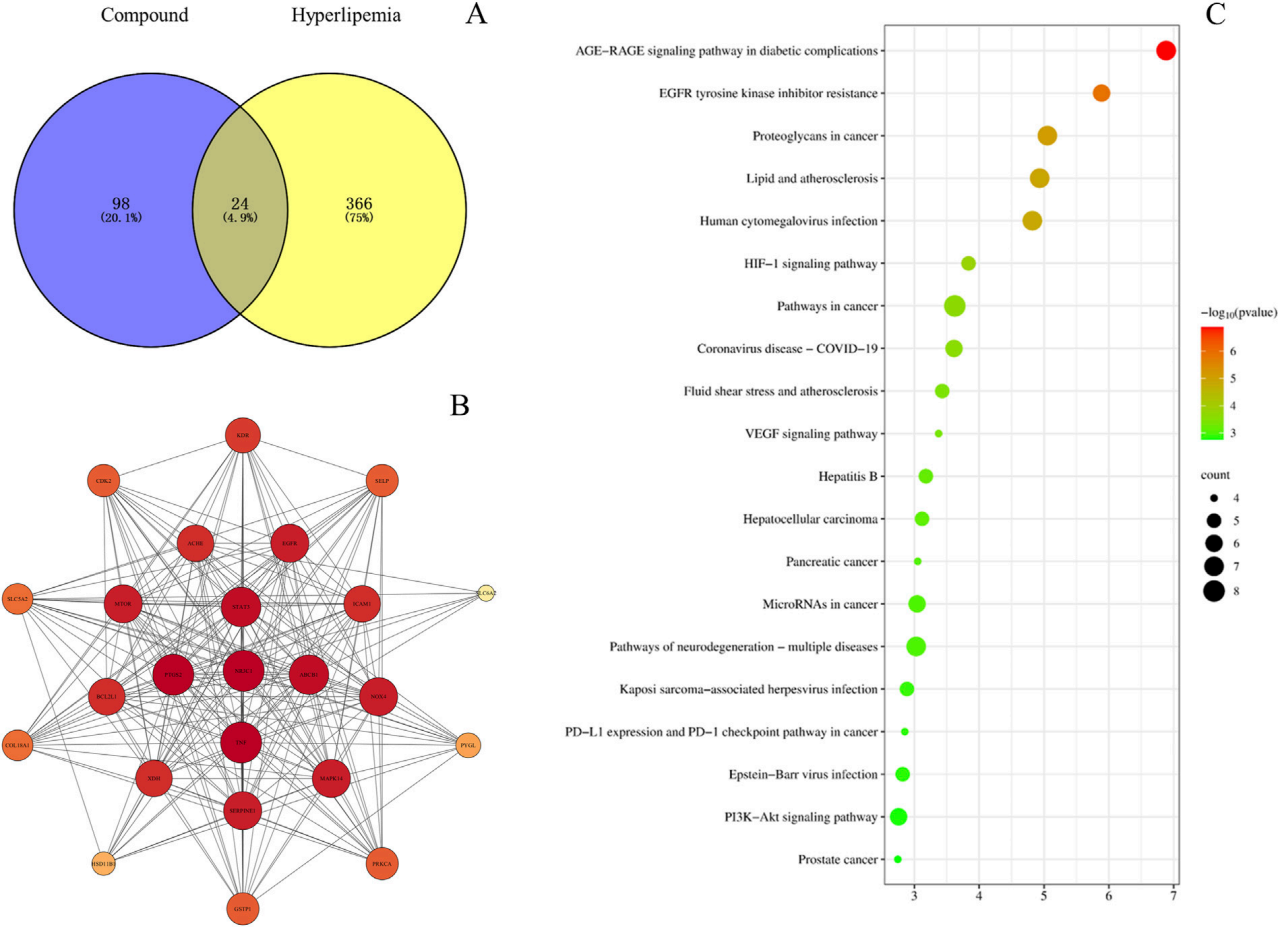
Venny diagram **(A)**, PPI network diagram **(B)**, and bubble diagram **(C)**.

The PK markers of BSTZC were found in 123 related targets by Swiss Target Prediction databases. The nine components are shown in Supplementary Figure S2. In addition, 390 HLP-related targets were indicated in CTD, GeneCards, DisGeNET, and Drugbank databases. Furthermore, 24 common targets were confirmed by Venny 2.1.0 (Figure 6A), including PTGS2, TNF, NR3C1, ABCB1, STAT3, SERPINE1, MAPK14, NOX4, EGFR, MTOR, ICAM1, ACHE, BCL2L1, XDH, KDR, SELP, CDK2, GSTP1, PRKCA, SLC5A2, COL18A1, PYGL, HSD11B1, and SLC6A2, and then overlapped targets were imported into String and Cytoscape 3.7.1 software to obtain the PPI network, as shown in Figure 6B. The results of the top 20 of the KEGG enrichment pathways are shown in the bubble diagrams in Figure 6C. The results of the KEGG enrichment pathway analysis mainly included lipid, and atherosclerosis pathways, cancer-related pathways, and virus infection. AGE-RAGE signaling pathway, lipid and atherosclerosis, HIF-1 signaling pathway, fluid shear stress and atherosclerosis, VEGF signaling pathway, and PI3K-Akt signaling pathway were relative to the treatment of HLP.

### 3.4 Results of lipid-lowering experiments with active components

**FIGURE 7:**
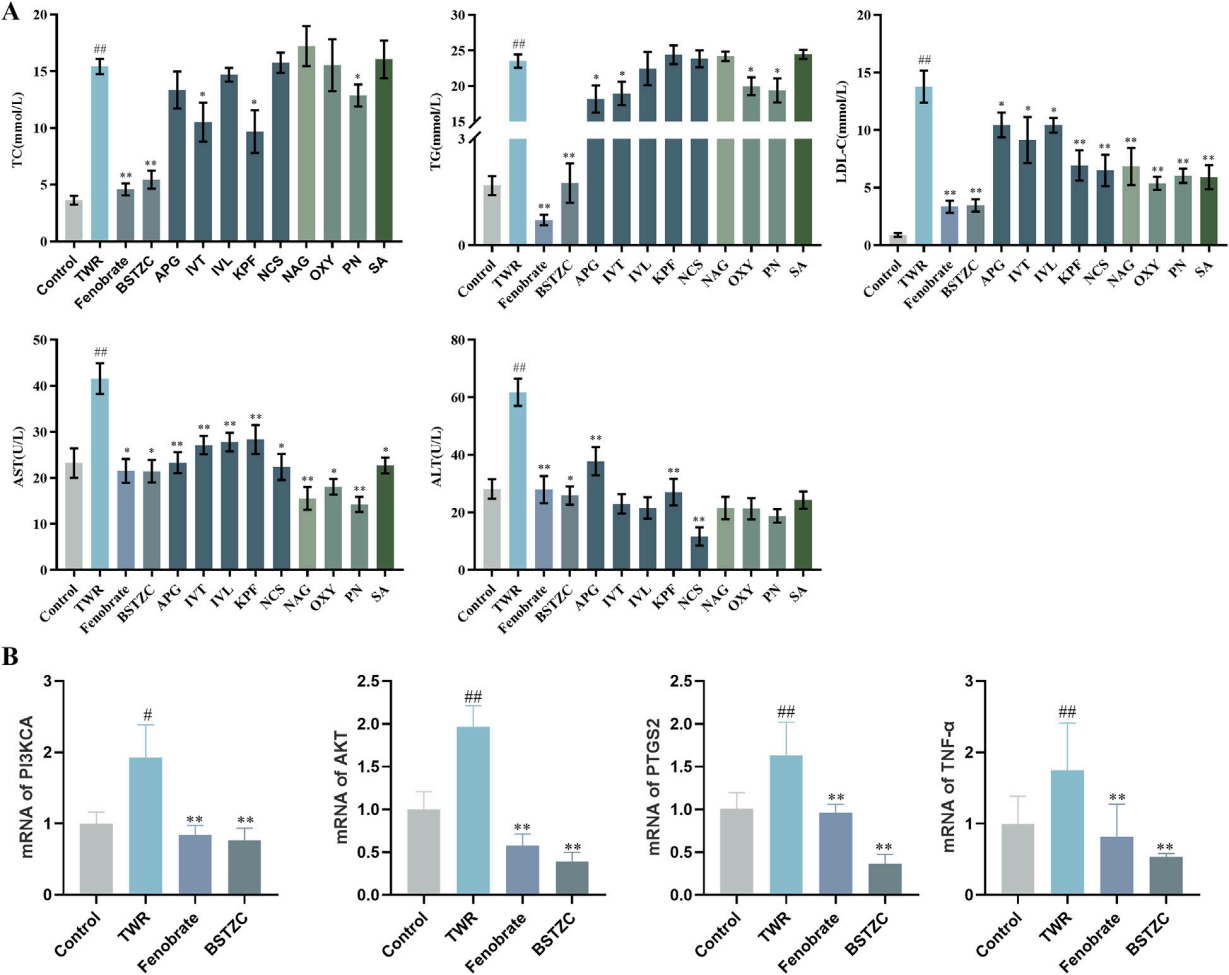
Lipid-lowering effect of BSTZC and its active components on triton WR-1339-induced HLP mice. **(A)** Biochemical analyses of serum TC, TG, LDL-c, ALT, and AST. **(B)** mRNA levels of PI3KCA, AKT, PTGS2, and TNF in mice liver. The results are represented as mean ± SD of six mice in each group. ^##^ *p* < 0.01 vs. the control group, **p < 0.01 < 0.01 vs. the model group.

The experimental results showed that the serum levels of TC, TG, LDL-C, ALT, and AST were significantly increased in the model group compared with the control group, indicating that the HLP model was successfully established (Figure 7A). After administration, the monomer components played different degrees of roles in lipid-lowering, compared with the model group, and the KPF group significantly reduced the serum levels of TC, LDL-C, ALT, and AST. The IVT group significantly downregulated the serum levels of TC, TG, LDL-C, and AST. The APG group significantly reduced the serum levels of TG, LDL-C, ALT, and AST. The PN group significantly reduced the levels of TC, TG, LDL-C, and AST in serum, suggesting that these components have good lipid-lowering and liver-protecting effects and that these components may be the active components in the lipid-lowering efficacy of BSTZC. In addition to the above components with lipid-lowering effects, other blood-absorbed components play a protective role for the liver to a certain extent by lowering LDL-C and AST in serum. Among them, the lipid-lowering efficacy of the TCM formula was obvious, and the administration of BSTZC effectively lowered the levels of TC, TG, LDL-C, ALT, and AST in serum, indicating that it could significantly improve the abnormalities of lipid metabolism and liver injury.

### 3.5 Effects of BSTZC on the PI3K/Akt signaling pathway

Combined with the core targets and pathways predicted by the absorption into the blood component network analysis, PTGS2 and TNF may be the key targets of BSTZC, and the ameliorating effect of BSTZC on HLP may be related to the PI3K/Akt signaling pathways. To validate this result, we performed RT-qPCR assays to determine the effect of BSTZC intervention on this pathway. As shown in Figure 7B, compared with the control group, the mRNA levels of AKT, PIK3CA, PTGS2, and TNF were significantly elevated in the TWR model group, and these key targets were significantly reversed after BSTZC treatment. These findings suggested that BSTZC may ameliorate HLP by inhibiting the PI3K/AKT signaling pathways.

## 4 Discussion

BSTZC is a novel Chinese medicine prepared for the treatment of HLP, which can reduce the risk of hyperlipidemia, atherosclerosis, and coronary heart disease, but there was still insufficient pharmacodynamic substance basis study. Therefore, an effective and quick screening method for active components of BSTZC was established in this work.

The serum pharmacochemistry revealed nine migrating metabolites in rat serum were determined, including five flavonoids, one organic acid, two monoterpenoids, and one diterpene lactone. The migrating metabolites had a certain concentration in the rat blood and high oral bioavailability, which was unambiguously identified by comparing their accurate mass measurements of MS and retention times to reference components. Flavonoids are antioxidant components that can prevent lipid oxidation, especially oxidative damage caused by HLP (Huang et al., 2020). Paeoniflorin and other monoterpenoids can protect the liver by activating AMPK and inhibiting lipid synthesis in hepatocytes (Li, Y.C. et al., 2018). Kaempferol and apigenin have an effect on lipid regulation and antioxidants, which can reduce the level of TC, TG, and MDA, and increase the activities of SOD and GSH-Px in serum (Ochiai et al., 2021; Ren et al., 2016). Furthermore, kaempferol has potential health benefits, which are related to cardiovascular protective mechanisms, such as anti-aging and cancer-preventive activities (Dabeek and Marra, 2019). However, both the two components were affected by UDP-glucuronosyltransferases (UGT) (Chen et al., 2008), promptly absorbed through the gastrointestinal tract, and metabolized to phase Ⅱ conjugates (Wang et al., 2014; Zhang et al., 2010). The metabolites were more rapidly eliminated due to the polarity enhancement. The metabolites were spread throughout various botanical drugs in BSTZC prescription (namely, monarch, minister, assistant, and guide), which confirmed each botanical drug was well absorbed. Nevertheless, the metabolites of Curcumae Rhizoma were undetected in rat blood probably because Curcumae Rhizoma contains mainly volatile components, and the next plan was to detect the Curcumae Rhizoma components in blood of rats using GC-MS (Li, W. et al., 2018).

HLP is a chronic systemic metabolic syndrome that becomes a risk factor for atherosclerosis and cardiovascular disease (Barness et al., 2007; Ng et al., 2014). Therefore, we generated the HFD-based rat model to investigate the dynamic changes of BSTZC in rats, compared with the healthy rats. The comparison of the two groups can give a theoretical foundation for clinical administration. The results of pharmacokinetics revealed that the T_max_ of all the components was within 3 h, which indicated that all the analytes were rapidly absorbed into the blood. Relative to rats in the control groups, those in the model group exhibited significantly increased T_max_, C_max_, and AUC values for all analytes, which might be attributed to physiological variables. In the pathological condition of HLP, gastrointestinal motility was impaired, leading to prolonged retention time, delayed absorption, and decreased excretion rates (Tong et al., 2012). Based on these cases, the total accumulation in the plasma, especially the flavonoids (Ying et al., 2020), was increased, including the IVT, IVL, and KPF-3-G. The T_max_ of kaempferol was approximately 2 h, whereas KPF-3-G reached the maximum concentration faster, and its t_1/2_ was prolonged, as the metabolite was kaempferol. KPF-3-G can reduce lipid accumulation and the level of reactive oxygen species through the Nrf2/Keap1 pathway (Deng et al., 2021). As the concentration–time profiles show, there was double-peak of KPF-3-G in the control group. The appearance of the double-peak phenomenon may be related to enterohepatic circulation, dual-site absorption, or intestinal efflux (Song et al., 2023). Similarly, we can observe the double-peak phenomenon of *p*N. The pharmacokinetic parameters of PN, as in prior studies, were significantly different in the single drug (Xu et al., 2016), such as T_max_ value 10.00 ± 1.73 min and t_1/2_ value 142.98 ± 30.11 min. Inconsistent with results from prior studies (Luo et al., 2014), the value of T_max_ and t_1/2_ were increased, as well as the residence time in rats was prolonged. PN was orally administrated to HLP model rats, which can activate AMPK, and downregulate the liver fat synthesis pathway, with the appearance of the rising level of APN in the serum, resulting in lipid metabolism acceleration and then having an effect on NAFLD treatment (Ma, Z. et al., 2017). Due to the interaction between components in TCM, the prolonged MRT promoted a therapeutic effect. Moreover, NCS can play an anti-diabetic role by regulating the key target proteins of PTGS2 and TNF, acting on metabolic pathways, and reducing the release of macrophage inflammatory factors to alleviate inflammation (Liu et al., 2023). APG can improve atherosclerosis by stimulating apoptosis of macrophages and downregulating the secretion of cytokines (TNF-α, IL-1β, and IL-6) (Clayton et al., 2021). The blood concentration of NCS and APG increased first and then decreased after oral administration.

To further understand the effectiveness of these nine components of BSTZC in the treatment of HLP, network analysis was an approach to predict the therapeutic pathways. The PPI network showed that common targets including PTGS2, TNF, and ABCB1 were probably the relevant targets for BSTZC in HLP treatment. It was found in these enriched pathways that the common signaling pathways were the HIF-1 signaling pathway, VEGF signaling pathway, and PI3K-Akt signaling pathway associated with the treatment of HLP. HIF-1 is an important transcription factor for hypoxic response, composed of HIF-1α and HIF-1β subtypes. HIF-1 signaling pathway and the protein expression were mediators, resulting in increased vascular endothelial permeability (Thomas et al., 2022). The release of inflammatory mediators leads to increased vascular permeability and angiogenesis in adipose tissue (Manalo et al., 2005). The PI3K-Akt signaling pathway mainly participates in regulating many biological processes, such as cell proliferation, differentiation, migration, and apoptosis (Vivanco and Sawyers, 2002). The risk of atherosclerosis, hypertension, and other cardiovascular diseases was efficiently decreased by suppressing the PI3K-Akt signaling pathway (Shao et al., 2018). NO is a vasodilator secreted by vascular endothelium, which can regulate vascular endothelial function and promote vascular endothelial regeneration and platelet adhesion. The phosphorylation of eNOS was promoted with stimulated Akt so that NO was generated to protect vascular endothelial from dysfunction (Lee et al., 2021). In addition, inhibition of the PI3K/Akt pathway can significantly downregulate LPL expression in macrophages, reduce lipid uptake by macrophages, reduce intracellular lipid accumulation, and delay cell foam formation (Sato et al., 2018). *In vivo* experiments showed that the lipid-lowering effect of BSTZC was obvious, and compared with the model group, BSTZC could effectively reduce the levels of TC, TG, LDL-C, ALT, and AST in mice serum, suggesting that it could alleviate the abnormalities of lipid metabolism and liver injury. Meanwhile, the lipid-lowering effects of APG, KPF, IVT, and PN groups were more obvious in each component group, suggesting that they may serve as the pharmacodynamic substance basis of BSTZC. Interestingly, the lipid-lowering effect of BSTZC was better than that of the single component, which fully reflected the multi-component and multi-target characteristics of BSTZC in the treatment of hyperlipidemia (Zhang et al., 2024). In addition, our study found that BSTZC inhibited the mRNA levels of PI3KCA, AKT, TNF, PTGS2, and TNF, suggesting that BSTZC may alleviate HLP by inhibiting the PI3K/AKT signaling pathway, further validating the results of the network analysis.

In conclusion, in this study, we mainly investigate the active components of BSTZC in treating HLP and verified the lipid-lowering effects of the active components through *in vivo* experiments, which provided the basis for the study of the pharmacological material basis and quality control system of BSTZC. In addition, BSTZC significantly alleviated lipid metabolism disorders and liver injury in the triton WR-1339-induced HLP mice model, and its mechanism may involve the regulation of the PI3K/AKT signaling pathway in the liver to exert lipid-lowering efficacy. In addition, because most preclinical studies have been conducted in male rodents, there are limitations in the present study in that only male animals were selected first, taking into account the issues of sex hormone effects, stability of experimental results, and efficiency of model building (Beery and Zucker, 2011; Cahill, 2006). Sex differences in metabolic traits such as obesity, diabetes, and cardiovascular disease have been well described in mice, humans, and other species, with females typically exhibiting more beneficial metabolic traits (Chella Krishnan et al., 2018). As the prevalence of lipid metabolism disorders is closely related to age and sex, female mice have relatively high estrogen levels, which has a protective effect and reduces the accumulation of lipids and the development of atherosclerosis. The postmenopausal condition is associated with a high prevalence of many features of the metabolic syndrome, including obesity, steatosis, and oxidative damage to the liver (Hermoso et al., 2016; Yu et al., 2021). In addition, male rats are more likely to develop obesity and metabolic diseases induced by a high-fat diet, and male rats are more sensitive to the catabolic effects of insulin, whereas female rats are more sensitive to the catabolic effects of anorexic leptin, which is determined by the central effect of estrogens. Moreover, female rats showed later diet-induced weight gain and fewer metabolic complications than male rats (Maric et al., 2022). Therefore, taking into account the sex difference, this study will also be conducted in the future to study the sex difference accordingly, and the lipid-lowering effect of BSTZC through this signaling pathway will be confirmed *in vivo* experiments using agonists of the PI3K/AKT signaling pathway.

## 5 Conclusion

In this work, an effective strategy was developed by integrating serum pharmacological chemistry, pharmacokinetics, network analysis, and experimental validation to explore the active components of BSTZC in the treatment of HLP. In this study, we explored the dynamics of nine migratory components as PK markers by comparing them with normal rats and HLP model rats. In addition, we found that BSTZC and its active components could ameliorate lipid abnormalities and liver injury, and BSTZC could alleviate HLP by modulating the PI3K/Akt pathway. In this study, we provide a systematic approach to explore the active components of TCM and provide useful information to guide the clinical application of BSTZC in treating HLP.

## Data Availability

The original contributions presented in the study are included in the article/Supplementary Material, further inquiries can be directed to the corresponding authors.

## Abbreviations

BSTZC, BuShao Tiaozhi Capsule; HLP, hyperlipidemia; IVT, isovitexin; IVL, isoviolanthin; NCS, narcissoside; OXY, oxypaeoniflorin; PN, paeoniflorin; SA, salicylic acid; NAG, neoandrographolide; KPF-3-G, kaempferol-3-glucuronide; APG-7-G, apigenin-7-glucuronide; KEGG, Kyoto Encyclopedia of Genes and Genomes data obtained; PK, pharmacokinetics; QC, quality control; LLOQ, lower limit of quantification; PPI, protein–protein; T_max_, the time to reach the maximum concentration; t_1/2_, terminal elimination half-life; C_max_, maximum concentration; AUC_0→t_, area under the concentration-time curve from zero to t; AUC_0→∞_, area under the concentration–time curve from zero to infinity; MRT, mean retention time; TC, cholesterol; TG, triglycerides; LDL, low-density lipoprotein; HDL, high-density lipoprotein; TCM, tradition Chinese medicine.
