# Supplementary material for: Integrated serum pharmacochemistry, pharmacokinetics, and network analysis to explore active components of BuShao Tiaozhi Capsule on hyperlipidemia
Source: Front Pharmacol. 2025 Jan 3;15:1444967. doi: 10.3389/fphar.2024.1444967 (PMC11738623; doi:10.3389/fphar.2024.1444967)
Supplement: Supplementary file 1 [file DataSheet1.docx]

**Table S1**. The information of the reference substances

| **Reference substances** | **Lot number** | **Purity** | **Supplier company** |
| --- | --- | --- | --- |
| Schaftoside | 111912-201703 | 95.6% | National Institutes for Food and Drug Control |
| Isorhamnetin 3-*O*-neohesperidoside | 111571-201205 | 93.20% |  |
| Narcissoside | 111997-201501 | 93.10% |  |
| Paeoniflorin | 110736-201943 | 95.10% |  |
| Curdione | 111800-201302 | >98.0% |  |
| Germacrone | 111665-201204 | >98.0% |  |
| Dehydroandrographolide | 111854-201007 | 99.70% |  |
| Vitexin | M-023-120429 | >98.0% | Chengdu Herbpurify CO.,LTD |
| Isovitexin | Y-116-120630 | >98.0% |  |
| Typhaneoside | X-020-120305 | >98.0% |  |
| Apigenin | Q-002-161216 | >98.0% |  |
| Neochlorogenic acid | X-014-180410 | >98.0% |  |
| Cryptochlorogenic acid | Y-067-180425 | >98.0% |  |
| Albiflorin | S-011-180908 | >98.0% |  |
| Apigenin-7-glucuronide | Q-072-180601 | >98.0% |  |
| Neoandrographolide | DSTDX000401 | >98.0% | Chengdu Biopurify Phytochemicals Ltd. |
| Oxypaeoniflorin | DST220707-074 | >98.0% |  |
| Tiliroside | PRF9101501 | >98.0% |  |
| Luteolin-3-*O*-beta-D-glucuronide | DST221019 | >98.0% |  |
| Salicylic acid | DSTDS012401 | >98.0% |  |
| Kaempferol | LMT0088-01 | >98.0% |  |
| Kaempferol-3-beta-*O*-glucuronide | DSTDS017501 | >98.0% |  |
| Sulfamethoxazole | DSTD200226-220 | >98.0% |  |
| Isoquercitrin | AF7070301 | >98.0% | Chengdu Alfa Biotechnology Co.Ltd. |
| Violanthin | AF802612 | >98.0% |  |
| Isoviolanthin | AF802613 | 95% |  |

**Table S2.** Parameters of mass spectrometry of the nine analytes and IS.

| **Compound** | **Precursor Ion (*m/z*)** | **Product Ion (*m/z*)** | **CE (V)** |
| --- | --- | --- | --- |
| OXY | 495.1 | 137.0 | 30 |
|  |  | 465.2 | 22 |
| PN | 479.1 | 449.2 | 6 |
|  |  | 120.8 | 18 |
| IVT | 431.1 | 311.0 | 20 |
|  |  | 283.1 | 38 |
| IVL | 577.1 | 383.2 | 42 |
|  |  | 352.9 | 40 |
| SA | 137.0 | 93.1 | 16 |
|  |  | 65.0 | 34 |
| KPF-3-G | 461.0 | 285.0 | 20 |
|  |  | 228.7 | 44 |
| NCS | 623.1 | 314.9 | 34 |
|  |  | 313.9 | 38 |
| APG-7-G | 445.0 | 269.1 | 22 |
| NAG | 525.2 | 479.1 | 12 |
| Sulfamethoxazole（IS） | 251.9 | 156.0 | 12 |

**Table S3.** The primer sequence formation.

| **Gene name** | **Forward primer (5’-3’)** | **Reverse primer (5’-3’)** |
| --- | --- | --- |
| TNF | CCCTCACACTCACAAACCACC | CTTTGAGATCCATGCCGTTG |
| AKT | GTCATCGAACGCACCTTCCAT | AGCTTCAGGTACTCAAACTCGT |
| PI3KCA | AGTAGGCAACCGTGAAGAAAAG | GAGGTGAATTGAGGTCC |
| PTGS2 | TACTCACAGTGCGGTCCAAC | TTGGGCCAGAAGCTGAACAT |
| GAPDH | GGAGCGAGATCCCTCCAAAAT | GGCTGTTGTCATACTTCTCATGG |

**Table S4.** Similarity evaluation of BSTZ fingerprints for 10 batches.

| **S1** | **S2** | **S3** | **S4** | **S5** | **S6** | **S7** | **S8** | **S9** | **S10** | **Reference** |
| --- | --- | --- | --- | --- | --- | --- | --- | --- | --- | --- |
| 1.000 | 1.000 | 0.994 | 0.994 | 0.997 | 0.997 | 0.998 | 0.998 | 0.993 | 0.992 | 0.999 |
| 1.000 | 1.000 | 0.994 | 0.994 | 0.997 | 0.997 | 0.998 | 0.998 | 0.993 | 0.992 | 0.999 |
| 0.994 | 0.994 | 1.000 | 1.000 | 0.986 | 0.986 | 0.997 | 0.997 | 0.983 | 0.990 | 0.996 |
| 0.994 | 0.994 | 1.000 | 1.000 | 0.986 | 0.986 | 0.997 | 0.997 | 0.983 | 0.990 | 0.996 |
| 0.997 | 0.997 | 0.986 | 0.986 | 1.000 | 1.000 | 0.995 | 0.995 | 0.995 | 0.986 | 0.996 |
| 0.998 | 0.997 | 0.986 | 0.986 | 1.000 | 1.000 | 0.995 | 0.995 | 0.995 | 0.986 | 0.996 |
| 0.998 | 0.998 | 0.997 | 0.997 | 0.995 | 0.995 | 1.000 | 1.000 | 0.991 | 0.992 | 0.999 |
| 0.998 | 0.998 | 0.997 | 0.997 | 0.995 | 0.995 | 1.000 | 1.000 | 0.991 | 0.992 | 0.999 |
| 0.993 | 0.993 | 0.983 | 0.983 | 0.995 | 0.995 | 0.991 | 0.991 | 1.000 | 0.992 | 0.994 |
| 0.992 | 0.992 | 0.990 | 0.990 | 0.986 | 0.986 | 0.992 | 0.992 | 0.992 | 1.000 | 0.994 |
| 0.999 | 0.999 | 0.996 | 0.996 | 0.996 | 0.996 | 0.999 | 0.999 | 0.994 | 0.994 | 1.000 |
| 1.000 | 1.000 | 0.994 | 0.994 | 0.997 | 0.997 | 0.998 | 0.998 | 0.993 | 0.992 | 0.999 |
| 1.000 | 1.000 | 0.994 | 0.994 | 0.997 | 0.997 | 0.998 | 0.998 | 0.993 | 0.992 | 0.999 |

**Table S5.** Chemical compositional identification and analytical results of BSTZC extracts and blood absorption components.

| **No.** | **Identification** | **Formula** | **MS（*m/z*）** | **Error(ppm)** | **MS^n^（*m/z*）** |
| --- | --- | --- | --- | --- | --- |
| 1 | Succinic acid | C_4_H_6_O_4_ | 117.0193[M-H]^-^ | -0.9 | 99.925 9, 83.929 8, 73.029 3 |
| 2 | Gallic acid | C_7_H_6_O_5_ | 169.014[M-H]^-^ | -1.2 | 125.024 2,124.016 1, 81.034 2, 79.018 3 |
| 3 | Protocatechuic acid | C_7_H_6_O_4_ | 153.0193[M-H]^-^ | -0.9 | 109.029 7, 108.021 5, 91.018 4, 81.034 5 |
| 4 | Neochlorogenic acid^*^ | C_16_H_18_O_9_ | 353.0877[M-H]^-^ | -0.4 | 191.056 3, 179.035 2, 173.045 1, 161.025 4, 135.044 9 |
| 5 | Oxypaeoniflorin ^*^ *^p^* | C_23_H_28_O_12_ | 541.1572[M+HCOO]^-^ | 1.8 | 495.150 7, 137.024 7, 465.142 7 |
| 6 | Chlorogenic acid | C_16_H_18_O_9_ | 353.0878[M-H]^-^ | 0.2 | 191.056 5, 179.035 0, 173.045 3, 135.044 8 |
|  |  |  | 355.1024[M+H]^+^ | 0.6 | 267.000 4, 163.039 5, 145.028 8, 89.038 8 |
| 7 | Catechin | C_15_H_14_O_6_ | 289.0718[M-H]^-^ | -1.0 | 221.083 1, 203.071 9, 175.077 3, 121.030 6 |
|  |  |  | 291.0863[M+H]^+^ | -0.1 | 207.065 7, 139.039 3, 123.044 5 |
| 8 | Cryptochlorogenic acid^*^ | C_16_H_18_O_9_ | 353.0876[M-H]^-^ | -0.5 | 191.054 9, 179.034 2, 173.046 0, 135.045 2 |
| 9 | Caffeic acid | C_9_H_8_O_4_ | 179.0351[M-H]^-^ | 0.9 | 135.045 2, 134.037 9, 107.050 9, 89.039 7, 79.055 8 |
| 10 | Epicatechin | C_15_H_14_O_6_ | 289.0718[M-H]^-^ | 0.1 | 245.081 3, 203.072 0, 165.019 6, 151.039 9, 109.029 7 |
|  |  |  | 291.0864[M+H]^+^ | 0.3 | 207.066 0, 139.039 0, 123.043 9 |
| 11 | Albiflorin ^*^ | C_23_H_28_O_11_ | 525.1614[M+HCOO]^-^ | 0.5 | 499.146 3, 479.155 8, 357.138 4, 273.076 4, 121.029 3 |
|  |  |  | 481.1704[M+H]^+^ | -0.4 | 319.118 8, 133.064 3, 105.032 5 |
| 12 | Schaftoside isomer | C_26_H_28_O_14_ | 563.1408[M+H]^+^ | 0.3 | 473.108 7, 443.100 5, 383.076 6, 353.068 3 |
| 13 | trans-4-hydroxycinnamic acid | C_9_H_8_O_3_ | 163.0399[M-H]^-^ | -0.8 | 145.029 4, 119.050 1, 93.034 6, 65.039 7 |
| 14 | Paeoniflorin^*^ *^p^* | C_23_H_28_O_11_ | 525.1615[M+HCOO]^-^ | 0.2 | 449.149 5, 479.157 0, 327.108 8, 121.028 9 |
| 15 | Mudanpioside E | C_24_H_30_O_13_ | 525.1614[M-H]^-^ | 0.2 | 193.051 0, 134.037 7 |
| 16 | Ferulic acid | C_10_H_10_O_4_ | 193.0508[M-H]^-^ | 0.8 | 178.027 1, 134.037 7, 133.029 6, 106.041 9, 77.038 8 |
|  |  |  | 195.0653[M+H]^+^ | 0.6 | 177.055 3, 145.028 7 |
| 17 | Schaftoside^*^ | C_26_H_28_O_14_ | 563.1406[M-H]^-^ | 1.7 | 473.108 6, 443.098 1, 383.076 6, 353.067 8, 325.071 7, 297.076 9 |
|  |  |  | 565.1552[M+H]^+^ | -1.2 | 409.092 5, 325.071 4 |
| 18 | Isoschaftoside ^*^ | C_26_H_28_O_14_ | 563.1409[M-H]^-^ | 0.4 | 503.119 8, 473.108 5, 443.098 1, 383.076 5, 353.068 3 |
|  |  |  | 565.1552[M+H]^+^ | -0.6 | 493.113 8, 379.082 6 |
| 19 | Salicylic acid *^p^ | C_7_H_6_O_3_ | 137.0243[M-H]^-^ | -0.6 | 93.034 5, 75.023 9, 65.039 9 |
| 20 | Ellagic acid | C_14_H_6_O_8_ | 300.9990[M-H]^-^ | 0.0 | 283.996 7, 245.010 2, 229.014 7, 185.023 9 |
|  |  |  | 303.0139[M+H]^+^ | 1.1 | 285.003 8, 275.019 1, 257.008 8 |
| 21 | Typhaneoside ^*^ | C_34_H_42_O_20_ | 769.2204[M-H]^-^ | 0.9 | 605.152 8, 314.044 0, 271.025 7 |
|  |  |  | 771.2344[M+H]^+^ | 0.2 | 317.066 0 |
| 22 | Rutin | C_27_H_30_O_16_ | 609.1478[M-H]^-^ | 2.8 | 301.034 5, 300.028 1, 283.028 1 |
|  |  |  | 611.1622[M+H]^+^ | 2.5 | 479.119 1, 317.066 2, 303.050 6 |
| 23 | Vitexin^*^ | C_21_H_20_O_10_ | 431.0984[M-H]^-^ | 0.1 | 341.065 5, 311.056 0, 283.051 3, 161.024 0 |
|  |  |  | 433.1128[M+H]^+^ | -0.3 | 415.102 2, 313.070 9, 283.051 3 |
| 24 | Isovitexin ^*^ *^p^* | C_21_H_20_O_10_ | 431.0984[M-H]^-^ | 0.1 | 341.063 8, 311.056 0, 283.061 3, 161.024 1, 117.033 9 |
|  |  |  | 433.1129[M+H]^+^ | -0.3 | 337.069 7, 313.070 9, 284.068 9 |
| 25 | Isoferulic acid | C_10_H_10_O_4_ | 193.0508[M-H]^-^ | 0.8 | 134.037 7, 132.021 5, 115.018 8, 106.041 9 |
|  |  |  | 195.0646[M+H]^+^ | -3.1 | 180.093 3, 149.060 0 |
| 26 | Violanthin ^*^ | C_27_H_30_O_14_ | 577.1566[M-H]^-^ | 0.6 | 559.145 2, 503.118 0, 383.075 5 |
|  |  |  | 579.1703[M+H]^+^ | -1.0 | 561.162 5, 543.150 8, 525.149 7 |
| 27 | Isoviolanthin ^*^ *^p^* | C_27_H_30_O_14_ | 577.1566[M-H]^-^ | 0.6 | 503.118 5, 383.075 8 |
|  |  |  | 579.1703[M+H]^+^ | -1.0 | 561.162 4, 543.1506, 525.140 8 |
| 28 | Isoquercitrin ^*^ | C_21_H_20_O_12_ | 463.0887[M-H]^-^ | 1.2 | 300.028 7, 271.024 8, 255.030 4 |
| 29 | Luteolin-7-*O*-glucoside | C_21_H_20_O_11_ | 447.0942[M-H]^-^ | 2.1 | 285.041 0, 255.028 8 |
|  |  |  | 449.1078[M+H]^+^ | 0.9 | 287.056 0, 153.018 8 |
| 30 | Luteolin-3-O-beta-D-glucuronide ^*^ | C_21_H_18_O_12_ | 461.0731[M-H]^-^ | 1.1 | 285.041 0 |
|  |  |  | 463.0871[M+H]^+^ | 0.7 | 287.055 3 |
| 31 | 6'-O-Galloyl paeoniflorin | C_30_H_32_O_15_ | 631.1676[M-H]^-^ | 1.2 | 313.057 8, 169.014 8 |
| 32 | 2-Carboxyphenol | C_7_H_6_O_3_ | 137.0244[M-H]^-^ | -0.6 | 93.034 5, 65.039 8 |
| 33 | Kaempferol ^*^ | C_15_H_10_O_6_ | 285.0403 [M-H]^-^ | 0.8 | 257.040 3, 241.055 0, 229.051 1 |
| 34 | Isochlorogenic acid B | C_25_H_24_O_12_ | 515.1195[M-H]^-^ | 2.2 | 353.088 8, 191.056 0, 173.045 8, 135,044 8 |
| 35 | Neohesperidin | C_28_H_32_O_16_ | 625.1756[M+H]^+^ | -1.1 | 317.065 5, 85.028 6 |
| 36 | Isorhamnetin 3-*O*-neohesperidoside^*^ | C_28_H_32_O_16_ | 623.1616[M-H]^-^ | -0.2 | 315.050 3, 314.042 5, 299.019 7, 271.024 9 |
| 37 | Astragalin | C_21_H_20_O_11_ | 447.0933[M-H]^-^ | 1.1 | 284.033 5, 255.028 9, 227.034 2 |
|  |  |  | 449.1085[M+H]^+^ | 1.6 | 287.055 6, 85.028 4 |
| 38 | Kaempferol-3-beta-*O*-glucuronide^*^ *^p^* | C_21_H_18_O_12_ | 461.0725[M-H]^-^ | 1.1 | 285.040 7, 257.043 3, 229.052 9 |
| 39 | Narcissoside^*^ *^p^* | C_28_H_32_O_16_ | 623.1618[M-H]^-^ | -0.2 | 357.061 8, 315.050 3 |
|  |  |  | 625.1771[M+H]^+^ | 1.3 | 479.117 2, 317.065 8, 302.042 6 |
| 40 | Isorhamnetin-3-*O*-beta-D-Glucoside | C_22_H_22_O_12_ | 477.1039[M-H]^-^ | 0.1 | 315.052 5, 285.041 2 |
|  |  |  | 479.1188[M+H]^+^ | 0.8 | 317.066 2, 302.043 3 |
| 41 | Apigenin^*^ | C_15_H_10_O_5_ | 269.0543[M-H]^-^ | 0.3 | 225.054 2, 151.003 6, 117.034 7, 107.014 5 |
| 42 | Apigenin-7-glucuronide^*^ *^p^* | C_21_H_18_O_11_ | 445.0776[M-H]^-^ | 1.9 | 269.046 3, 113.211 3, 59.013 6 |
| 43 | Luteolin-7-G-lucuronide | C_21_H_18_O_13_ | 477.0687[M-H]^-^ | 2.5 | 301.035 7 |
| 44 | Azelaic acid | C_9_H_16_O_4_ | 187.0978[M-H]^-^ | 0.9 | 143.108 8, 125.096 6, 97.065 6, 57.034 9 |
| 45 | Scutellarin | C_21_H_18_O_12_ | 461.0727[M-H]^-^ | 0.4 | 299.055 5, 285.040 1, 283.038 5 |
| 46 | neoprocurcumenol | C_15_H_22_O_2_ | 235.1693[M-H]^-^ | 0.1 | 175.148 5, 133.101 7 |
| 47 | Andrographolide | C_20_H_30_O_5_ | 349.2029[M+HCOO]^-^ | 0.0 | 331.192 0, 287.200 0, 108.020 9 |
|  |  |  | 351.2185[M+H]^+^ | 0.1 | 333.206 5, 315.196 2, 297.186 7, 285.186 3 |
| 48 | Luteolin | C_15_H_10_O_6_ | 287.0548[M+H]^+^ | 1.5 | 287.055 6, 165.018 6, 153.018 6 |
|  |  |  | 285.0405[M-H]^-^ | 0 | 201.019 5, 199.046 2, 151.003 6, 133.029 6 |
| 49 | Decanedioic acid | C_10_H_18_O_4_ | 201.1134[M-H]^-^ | 1.1 | 183.102 4, 139.112 7, 137.096 5, 111.081 6 |
| 50 | Tiliroside ^*^ | C_30_H_26_O_13_ | 593.1302[M-H]^-^ | 0.2 | 447.093 6, 285.040 8 |
|  |  |  | 595.1452[M+H]^+^ | 1.0 | 287.055 7, 147.044 5 |
| 51 | Isoandrographolide | C_20_H_30_O_5_ | 395.2077[M+HCOO]^-^ | 0.4 | 331.192 5, 287.201 8, 213.158 8 |
| 52 | Andropanoside | C_26_H_40_O_9_ | 541.2657[M+HCOO]^-^ | 0.4 | 495.260 9, 333.208 3 |
| 53 | 14-Deoxy-11,12-didehydroandrographiside | C_26_H_38_O_9_ | 539.2497[M+HCOO]^-^ | -0.1 | 493.244 3, 331.192 5, 101.024 5 |
| 54 | Benzoylpaeoniflorin | C_30_H_32_O_12_ | 629.1886[M+HCOO]^-^ | 1.5 | 553.173 0, 431.134 6, 121.029 8 |
| 55 | Isorhamnetin | C_16_H_12_O_7_ | 315.0512[M-H]^-^ | 0.5 | 300.028 1, 151.003 9 |
| 56 | Neoandrographolide ^*^*^p^* | C_26_H_40_O_8_ | 525.2701[M+HCOO]^-^ | -0.9 | 479.266 5, 317.213 4, 161.045 4 |
| 57 | Dehydroandrographolide ^*^ | C_20_H_28_O_4_ | 331.1915[M-H]^-^ | -0.1 | 303.191 7, 283.170 4, 255.175 2 |
| 58 | Curcumenol | C_15_H_22_O_2_ | 235.1693[M+H]^+^ | -0.6 | 217.1604, 189.164 0, 177.127 4, 161.096 4, 135.080 9 |
| 59 | Zederone | C_15_H_18_O_3_ | 247.1327[M+H]^+^ | -0.6 | 229.124 0, 201.128 0, 183.1183, 145.065 2, 139.039 0, 121.028 6 |
| 60 | Curdione^*^ | C_15_H_24_O_2_ | 237.1849[M+H]^+^ | -2.1 | 219.174 6, 191.180 4, 149.172 0, 133.116 8 |
| 61 | Furanogermacrene | C_15_H_20_O_2_ | 233.1535[M+H]^+^ | 0.3 | 215.144 1, 187.148 8, 172.1250, 145.101 9 |
| 62 | Isofuranodienone | C_15_H_18_O_2_ | 231.1377[M+H]^+^ | -1.2 | 213.128 1, 198.104 1, 173.096 5, 161.060 0 |
| 63 | Curcumenol | C_15_H_24_O_2_ | 237.1849[M+H]^+^ | 0.8 | 219.174 3, 201.164 3, 177.127 5 |
| 64 | Germacrone ^*^ | C_15_H_22_O | 219.1742[M+H]^+^ | -0.4 | 201.164 3, 177.127 8,  163.112 0, 159.117 6, 145.101 5, 117,076 9, 105.070 3 |

Annotation: * Identified by comparing with the standards; *^p^* components in rat serum after oral administration of BSTZC.

**
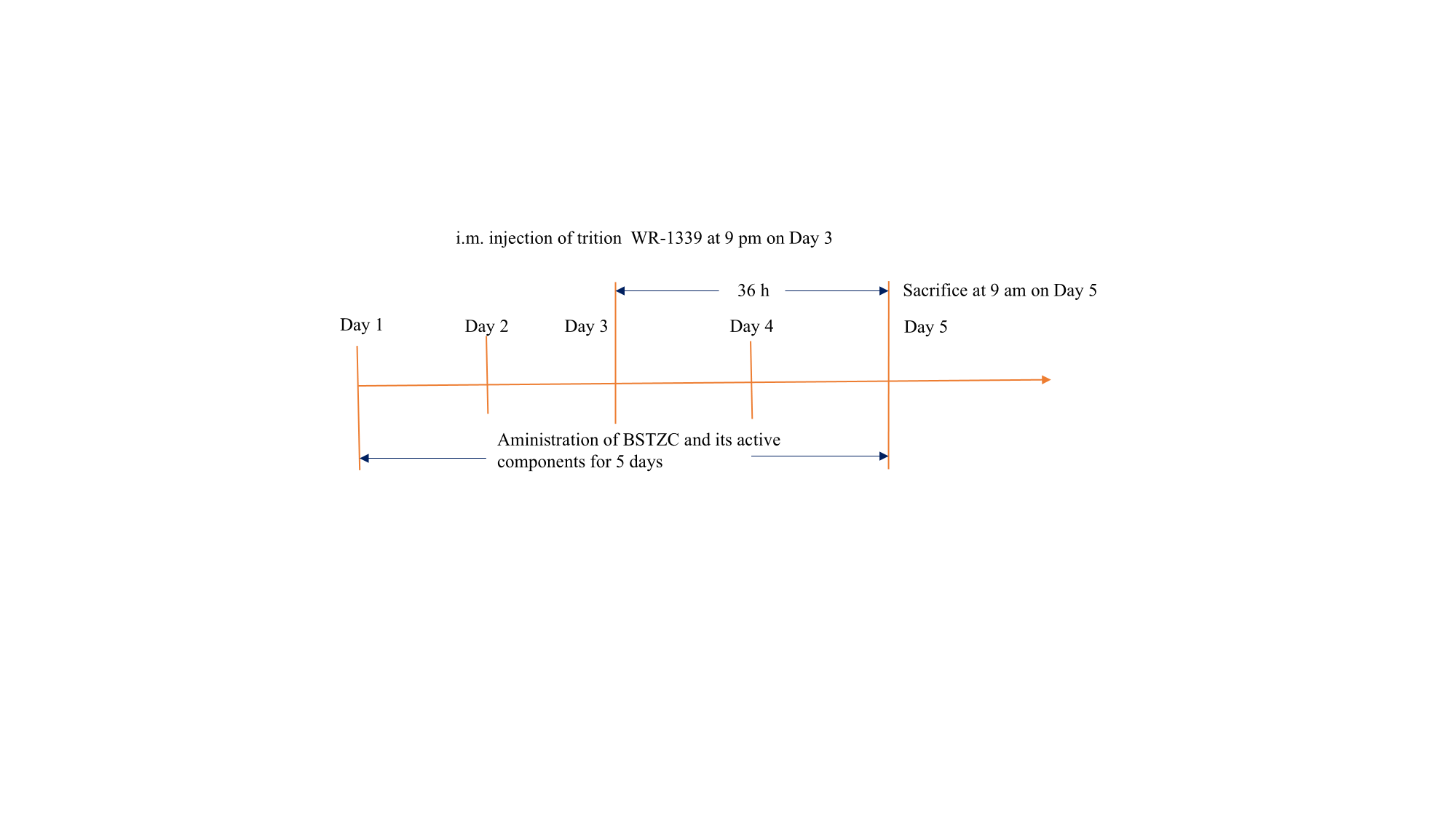
Figure S1.** Process of administration of BSTZC and its active components and HLP modeling.

**Figure S2.** The structural formulas of nine compounds.

**
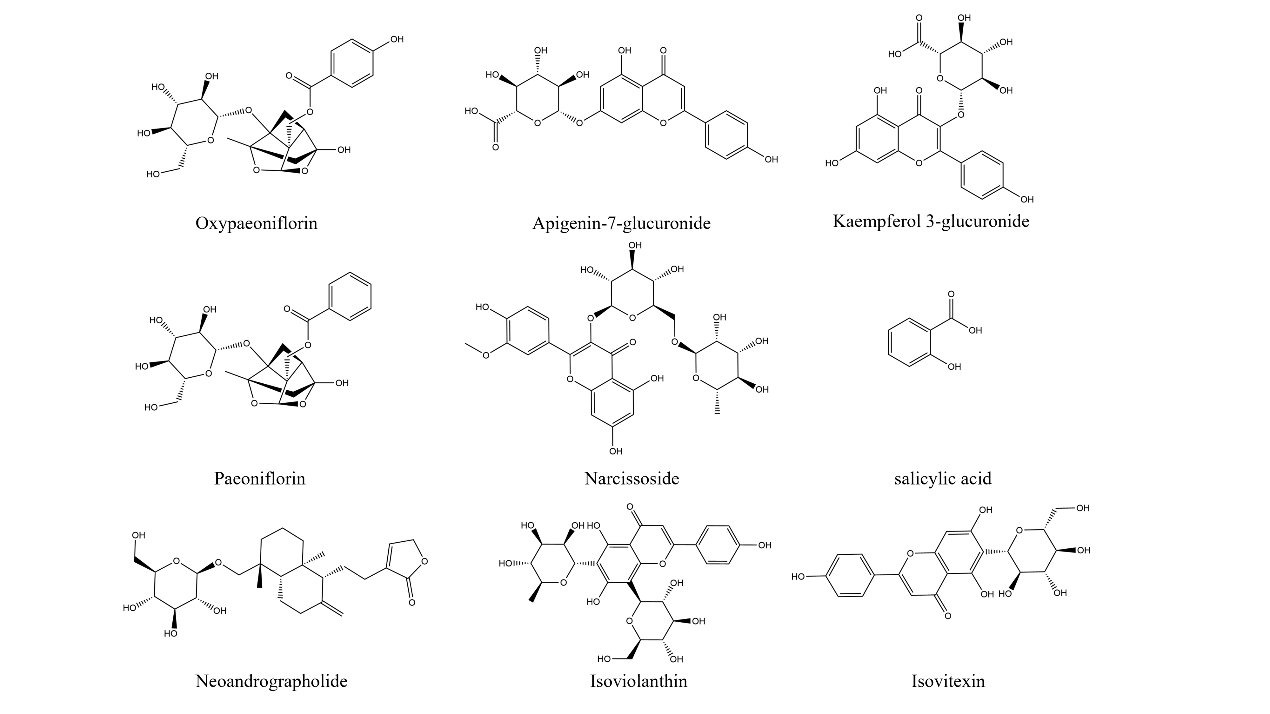
**
